# Generalized Surface Conductivity Model for Anisotropic Phonon Polaritons in van der Waals Slabs

**DOI:** 10.1002/nap2.70010

**Published:** 2026-01-20

**Authors:** Shuo Chen, Yuchen Sun, Jing Wu, Ceji Fu, Guangwei Hu

**Affiliations:** ^1^ LTCS School of Mechanics and Engineering Science Peking University Beijing China; ^2^ School of Electrical and Electronic Engineering Nanyang Technological University Singapore; ^3^ CINTRA (CNRS–International‐NTU‐THALES‐Research Alliances/UMI 3288) Singapore; ^4^ School of Electronic Science and Engineering Southeast University Nanjing China

**Keywords:** generalized surface conductivity model, higher‐order waveguide mode, phonon polaritons, photonic local density of states, van der Waals crystal

## Abstract

Recent advancements of anisotropic phonon polaritons (PhPs) in low‐dimensional van der Waals (vdW) materials enable efficient control of long‐wavelength light at nanoscale with ultrahigh confinement and low loss. The theoretical analysis based on the two‐dimensional (2D) surface conductivity model has been widely exploited, for its simplicity, to understand fundamental phenomena at the surface of vdW slabs, which, however, neglects the intrinsic higher‐order waveguide modes excited therein. Here, we report a generalized surface conductivity model which can allow us to include all waveguide modes, by taking into account the out‐of‐plane dimensions. In doing so, we can separate and examine each individual waveguide mode in vdW slabs with 2D models, and to further clarify the contribution of each polaritonic mode in near‐field light matter interactions. As a concrete example, we examine the enhancement of photonic local density of states by PhPs in the *α*‐phase molybdenum trioxide and hexagonal boron nitride plates and show that higher‐order waveguide PhPs, instead of fundamental ones, surprisingly dominate the enhancement of light–matter interactions close to the surface. Our findings provide fundamentally relevant insights into anisotropic polaritons in vdW materials and beyond, important in near‐field energy and information transport and other nanophotonic phenomena.

## Graphical abstract

Herein, we report a generalized surface conductivity model which includes all waveguide polariton branches, by taking into account the out‐of‐plane dimension. Using this generalized 2D model can separate and examine each individual polaritonic mode in van der Waals slabs and further clarify their contributions in near‐field light matter interactions.

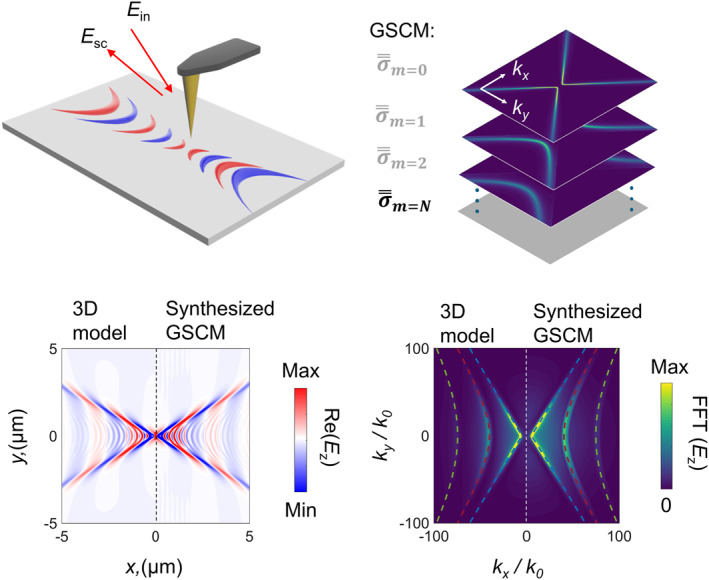

## 1 Introduction

Phonon polaritons (PhPs), that is, hybrid light–matter interaction modes arising from the strong coupling of photons and lattice vibrations, in emerging van der Waals (vdW) crystals exhibit the deep subwavelength confinement and ultralow loss propagation, offering new possibilities for long‐wavelength nanophotonics [1, 2, 3, 4, 5]. Particularly, PhPs in anisotropic vdW crystals [6, 7, 8] can show exotic topologies in their iso‐frequency contours (IFCs), with the reconfigurable photonic density of states and other propagation characteristics [9, 10]. Various promising applications have been explored such as enhanced thermal emission [11, 12, 13, 14], hyperspectral molecular sensing [15, 16, 17], and the nanoscale control of flow of light [18, 19, 20, 21, 22]. Among these technological developments, the accurate modeling of anisotropic PhPs in vdW crystals is of central importance.

**FIGURE 1:**
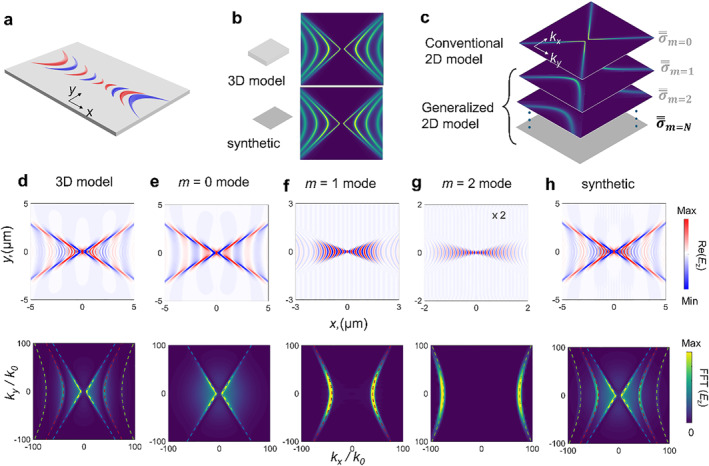
The generalized 2D model for biaxial vdW *α*‐MoO_3_ materials with 200 nm thickness. (a) Schematic diagram of hyperbolic polaritons excited by a vertical dipole in the vdW slab. (b) The dispersion of PhPs in the slab based on the 3D model ($\overset{═}{\varepsilon }$, top) and the synthetic dispersion as the superposition of individual modes based on the generalized 2D model ($\overset{═}{\sigma }$, bottom). (c) Illustration of the conventional and generalized 2D model. ${\overset{═}{\sigma }}_{m}$ represents the corrected conductivity for the *m*th order polaritonic mode excited in slab configuration. (d–h) Numerically simulated field distribution (real part of the *z*‐component of the electric field, Re(*E*_*z*_), top) and corresponding dispersion(FFT, bottom) based on 3D model (d) and the generalized 2D model (e–h), where the corrected conductivity ${\overset{═}{\sigma }}_{m=0}$, ${\overset{═}{\sigma }}_{m=1}$, and ${\overset{═}{\sigma }}_{m=2}$ are used for fundamental mode (e), *m* = 1 mode (f), and *m* = 2 mode (g) at 910 cm^−1^. The vertical electric dipole is 50 nm above the interface and the electric field at height of 20 nm is recorded. The dashed lines are the analytically calculated dispersion curves, among which the blue, red, and green lines represent the fundamental mode, *m* = 1 mode, and *m* = 2 mode, respectively. (h) Top: The synthetic electric field, namely, the superposition of *m* = 0, m = 1 and *m* = 2. Bottom: FFT of the top electric field map.

So far, two models are widely explored to understand anisotropic PhPs in vdW thin films, as shown in Figure 1a. The first is the fully rigorous three‐dimensional (3D) model where each layer carries its unique dielectric tensor ($\overset{═}{\varepsilon }$). This model can capture all polaritonic modes in the system, as shown in the top panel of Figure 1b. However, such method is computationally heavy, all modes obtained are non‐separable, and it needs more efforts to obtain physical insights therein. Another approach is to treat the vdW thin film as a two‐dimensional (2D) surface conductivity layer, $\overset{═}{\sigma }$ [23, 24, 25, 26, 27, 28]. This 2D model is simple and can capture various essential physics such as fundamental polaritonic modes with clear physical pictures like interlayer‐coupling‐induced hyperbolic‐to‐elliptic topological transitions in twisted vdW bilayers, which agrees well with the near‐field experiments [25]. We denote such 2D surface conductivity with fundamental modes as ${\overset{═}{\sigma }}_{m=0}$ (see the top panel of Figure 1c). However, the surface model by its nature fails to include the higher‐order polaritonic guided modes in the thin film, which may cause significant inaccuracies. Therefore, to overcome these limitations while retaining the advantages of 2D model, it is imperative to revisit the current $\overset{═}{\sigma }$ model to completely model PhP properties.

Here, we report a generalized surface conductivity model (GSCM) by taking into account the out‐of‐plane dimensional permittivity. This allows us to capture and separate all higher‐order modes, via the generalized 2D $\overset{═}{\sigma }$ model as ${\overset{═}{\sigma }}_{m=1}$, ${\overset{═}{\sigma }}_{m=2}$, $\text{…}{\overset{═}{\sigma }}_{m=N}$ (Figure 1c), while offering the full modeling of anisotropic PhPs with the matched dispersion in 3D $\overset{═}{\varepsilon }$‐models (see the bottom panel of Figure 1b). In doing so, we can calculate the polaritons of specific orders independently (Figure 1e–g) and synthetize them (Figure 1h) to obtain polariton patterns that are identical to 3D models (see Figure 1d). Based on our theoretical analysis and numerical verification, we reveal that the minor difference between the 3D model and the generalized model is associated with the inverse of the dielectric function along the out of plane direction. Moreover, the generalized 2D model enables us to separate each waveguided modes; using this feature, we explore the contribution from each polaritonic mode to the photonic local density of states (LDOS) enhancement.

The whole paper is organized as follows. We will first discuss the correction of the 2D $\overset{═}{\sigma }$‐model for in‐plane isotropic hyperbolic, with the example of hexagonal boron nitride (h‐BN), and then discuss the generalized surface model for in‐plane anisotropic case, illustrated by the biaxial *α*‐phase molybdenum trioxide (*α*‐MoO_3_) slab. We then show the dipole‐launched electric field distribution and the photonic LDOS to demonstrate the advantages of our method in separating different orders, which opens additional degree of freedom for dispersion engineer and thermal management at the nanoscale. Last, we conclude that the higher‐order mode dominates the contribution to photonic LDOS when the height is small.

## 2 Result and Discussion

### 2.1 The Correction for In‐Plane Isotropic 2D Model

To show our generalized surface conductivity model, we firstly consider an in‐plane isotropic material, taking h‐BN [29, 30, 31, 32, 33] thin film as the case study, which nevertheless can be extended for other materials and polaritonic system, including Be_2_Se_3_ [34, 35, 36], WSe_2_ [37], and SnO_2_ [38]. Here, the materials can be characterized as $\overset{═}{\varepsilon }=\text{diag}\left[\begin{array}{ccc} \varepsilon_{⊥} & \varepsilon_{⊥} & \varepsilon_{∥} \end{array}\right]$, where $\varepsilon_{⊥}$ and$\varepsilon_{∥}$ represent the permittivities along the direction perpendicular and parallel to the optic axis, respectively. This material will host the out‐of‐plane hyperbolic response $\varepsilon_{∥}\varepsilon_{⊥}<0$ for certain frequency ranges within the Reststrahlen band (RB). For simplicity, we consider the h‐BN slab (with the thickness *t*) suspended in air. The permittivities of h‐BN can be described by Lorentz model [29, 30, 33], whose details are provided in the Supporting Information S1. The dispersion relation for this h‐BN slab, with substrate and superstrate as the air, can be derived from Refs. [39, 40] and formulated as follows:

(1) $$\begin{array}{c} \sqrt{\frac{\varepsilon_{⊥}}{\varepsilon_{∥}}}+\varepsilon_{⊥}+\left(\sqrt{\frac{\varepsilon_{⊥}}{\varepsilon_{∥}}}-\varepsilon_{⊥}\right)e^{i\phi }=0 \end{array}$$

which is under the assumption of large wave vector.

Here, the infinite orders of PhPs modes can be found, whose dispersions, obtained from solving the source‐free Maxwell equations with 3D $\overset{═}{\varepsilon }$‐models, as follows:

(2) $$\begin{array}{c} e^{i\phi }=1+i\left(i\sqrt{\frac{\varepsilon_{⊥}}{\varepsilon_{∥}}}k_{\rho }t\pm m\pi \right),m=0,1,2\text{…} \end{array}$$

where t is the thickness of hBN thin slab. Substituting the Equation (2) into Equation (1), we can obtain the expression of dispersion relation as follows:

(3) $$\begin{array}{c} k_{\rho }=i\left(\frac{\text{sign}\left(\varepsilon_{⊥}\right)*2}{i\left(1-\sqrt{\varepsilon_{⊥}\varepsilon_{∥}}\right)}+m\pi \right)\frac{1}{t}\sqrt{\frac{\varepsilon_{∥}}{\varepsilon_{⊥}}} \end{array}$$

where sign(.) is the sign function, sign(x)=1 when x>0, and sign(x)=−1 when x<0. Namely, the expression closely depends on the sign of $\varepsilon_{⊥}$. With prior knowledge that for a simple surface conductivity, whose surface mode can be found as follows [41, 42]:

(4) $$\begin{array}{c} 2+\sigma \frac{ik_{\rho }}{\omega \varepsilon_{0}}=0, \end{array}$$

Comparing Equation (3) with Equation (4), we can obtain the generalized surface conductivity as follows:

(5) $$\begin{array}{c} \sigma =\frac{2\omega \varepsilon_{0}\sqrt{\frac{\varepsilon_{⊥}}{\varepsilon_{∥}}}t}{\pm \frac{2i}{\sqrt{\varepsilon_{⊥}\varepsilon_{∥}}}+m\pi }, \end{array}$$

where we simplify $\sqrt{\varepsilon_{⊥}\varepsilon_{∥}}-1$ as $\sqrt{\varepsilon_{⊥}\varepsilon_{∥}}$. The + corresponds to $\varepsilon_{⊥}>0$ (lower RB) and the—to $\varepsilon_{⊥}<0$ (upper RB). Here, we apply the first‐order approximation to linearize the dispersion relation in 3D case and retain mπ phase wrapping in Equation (2). In sharp contrast to previous studies [23, 24, 25, 26, 27], the phase wrapping term allows us to include higher‐order waveguide modes in the generalized 2D model. The surface conductivity is degraded into the conventional 2D model when *m* = 0, which validates our generalized 2D model. Besides, from Equation (5), one can see that the out‐of‐plane dimensional permittivity, that is, $\varepsilon_{∥}$ occurs in the corrected surface model of higher‐order modes, compared with the conventional surface model. Detailed derivation can be seen in Supporting Information S1: Section S10.

**FIGURE 2:**
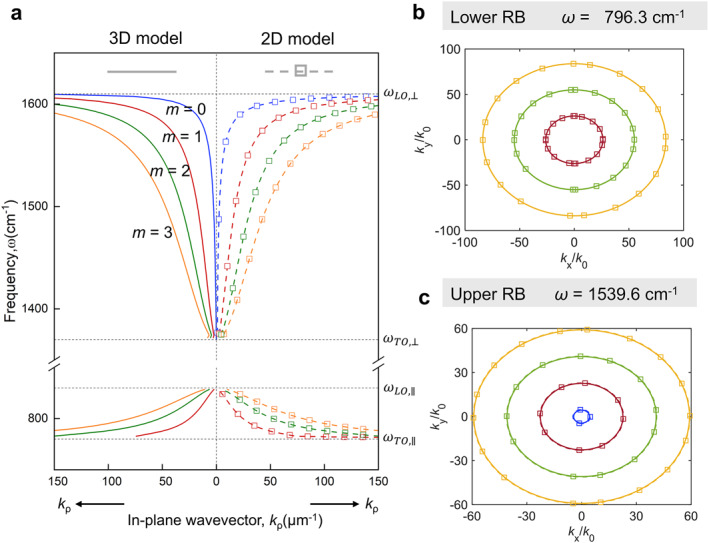
Diagrams showing the dispersion relation of a h‐BN plate calculated by 3D model and generalized 2D model. (a) Polariton frequency (*ω*)‐in‐plane wavevector (*k*_*ρ*_) dispersion relation. Due to the in‐plane isotropy of h‐BN, the dispersion in all azimuthal angles is the same. The dotted black lines represent the LO and TO phonon frequencies parallel and perpendicular to the optic axis, respectively. The IFCs at the frequency of 796.3 cm^−1^ (b) and 1539.6 cm^−1^(c), where the dispersion curves are circular‐like shapes. Thickness of the h‐BN plate is 200 nm. The solid curves represent the analytically calculated dispersion using the 3D model and the dashed ones are calculated by the generalized 2D models. The blue, red, green, and yellow curves denote the *m* = 0, *m* = 1, *m* = 2, and *m* = 3 modes, respectively.

The generalized 2D model achieves simplicity while preserving accuracy compared with 3D model. To demonstrate it, Figure 2 shows the dispersion contour of a suspended h‐BN slab with 200 nm thickness. Figure 2a reveals discrete dispersion curves in lower and upper RBs using both generalized 2D model (dashed line) and 3D model (solid line), which showcases that our generalized 2D surface conductivity model can correctly capture both fundamental and higher‐order modes. We further plot the IFCs of PhPs dispersions in the *k*_x_–*k*_y_ momentum space at lower RB (Figure 2b) and upper RB (Figure 2c), respectively. The analytical dispersion curves calculated from generalized 2D model agree excellently with the results adopting 3D model, further validating our generalized 2D model in polaritonic analysis.

**FIGURE 3:**
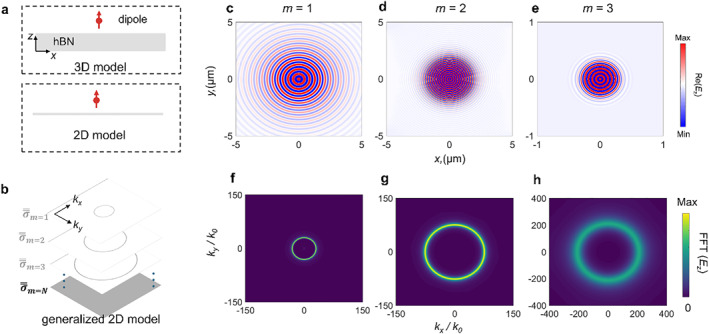
The generalized 2D model for the in‐plane isotropic h‐BN slab. (a) Schematic of the h‐BN slab, which can be modeled as 3D model (top) or 2D model (bottom). The PhPs are launched by vertically oriented electric point dipole sources placed on the top of structure at 796.3 cm^−1^. (b) Illustration of the generalized 2D model. ${\overset{═}{\sigma }}_{m}$ denotes the corrected conductivity tensor for the *m*th order polaritonic mode excited in slab configuration. Note that in lower RB of h‐BN, the lowest‐ordered mode is the *m* = 1 mode, hence *m* = 1, 2, …*N*. (c–e) Simulated Re(*E*_*z*_) via the generalized 2D model. The electric field distribution maps excited by the *m* = 1 mode (c), *m* = 2 mode (d), *m* = 3 mode (e) adopt the conductivity of ${\overset{═}{\sigma }}_{m=1}$, ${\overset{═}{\sigma }}_{m=2}$, and ${\overset{═}{\sigma }}_{m=3}$, respectively. Because of in‐plane isotropy, the electric field propagates omnidirectionally. (f–h) The corresponding dispersions (FFT). The thickness is 100 nm.

To further verify the generalized 2D model, we investigate the electric field excited by a vertical dipole as shown in Figure 3. Note that the conventional surface model is inherently dependent on the in‐plane permittivity, that is, *ε*_*x*_ and *ε*_*y*_. Only when either Re(*ε*_*x*_) or Re(*ε*_*y*_) is negative, or both are negative, can the surface mode be excited. In contrast, the surface mode is absent when the real parts of *ε*_*x*_ and *ε*_*y*_ are both positive, explaining the reason why the m=0 mode does not exist in the lower RB (Figure 2). Hence, it is essential to capture the higher‐order modes in 2D model, especially for uniaxial materials where the real parts of the in‐plane permittivity are both positive. Here, we focus on dipole‐launched electric field, where the h‐BN slab is modeled by 3D and generalized 2D models as shown in Figure 3a. Optic axis of h‐BN is oriented perpendicular to the horizontal slab surface, supporting the propagation of hyperbolic polaritons inside the h‐BN slab. The electric‐field distribution in *xz* plane is the superposition of contributions from all bulk modes. When using the generalized 2D model, each excited mode is modeled with individual conductivity, delineated by Figure 3b, where the lowest order is m=1 for the lower RB. Out‐of‐plane permittivity is taken into account, thus retaining the phase wrapping. The electric‐field distributions in *x‐y* plane as shown in Figure 3c–e, indicate in‐plane circular propagation of hyperbolic polaritons for uniaxial materials. As we expect, the corresponding FFT maps (Figure 3f–h) further corroborate the excitation of higher‐order modes. In comparison to bulk modes whose energy is confined inside h‐BN slab, the electric field of the higher‐order mode using 2D model is confined at the surface and propagates periodically along the surface due to the intrinsic surface properties. Namely, the waveguide bulk mode in the 3D model is transformed to surface mode in the frame of the 2D model, even though the out‐of‐plane evanescent waves decay in the same manner.

### 2.2 The Naturally High In‐Plane Anisotropy and Dispersion Analysis

More generally, we can extend our method to any kind of in‐plane anisotropic biaxial material. To demonstrate the generality, we investigate the dispersion of a highly non‐trivial case, *α*‐MoO_3_, using our generalized 2D model. The optical response of the *α*‐MoO_3_ is dominated by the phonon absorption, and its permittivity tensor components can also be described by the Lorentz model [23, 43]. The details of permittivity of *α*‐MoO_3_ are provided in Supporting Information S1. In comparison to h‐BN, *α*‐MoO_3_ supports anisotropic PhPs in three Reststrahlen bands: RB 1 in the range from 545 to 851 cm^−1^ and RB 2 in the range from 820 to 972 cm^−1^, in which the in‐plane IFC is hyperbolic; whereas an in‐plane dispersion of elliptical shape exists for PhPs in RB 3 (from 958 to 1010 cm^−1^). In a 3D model, with material's crystallographic directions parallel to axes in the Cartesian coordinate system, the general dispersion relation of *α*‐MoO_3_ reads as follows [27, 44, 45, 46, 47, 48]:

(6) $$\begin{array}{c} k_{\rho }=\frac{\rho }{t}\left[\arctan\left(\frac{\varepsilon_{1}\rho }{\varepsilon_{z}}\right)+\arctan\left(\frac{\varepsilon_{3}\rho }{\varepsilon_{z}}\right)+m\pi \right],m=0,1,2,3\text{…} \end{array}$$

where *t* is the thickness of *α*‐MoO_3_ thin slab, $k_{\rho }=\sqrt{k_{x}^{2}+k_{y}^{2}}$ is the in‐plane wavevector component, and $\rho =i\sqrt{\varepsilon_{z}/\left(\varepsilon_{x}\;\cos^{2}\;\varphi +\varepsilon_{y}\;\sin^{2}\;\varphi \right)}$ with *φ* being the angle between the *x*‐axis and *k*_*ρ*_, that is, propagation direction of the polaritons.

In the conventional 2D model, the dispersion relation of the anisotropic surface can be simplified under large wavevector approximation, $k_{x}^{2}+k_{y}^{2}-k_{0}^{2}\approx k_{x}^{2}+k_{y}^{2}$, and the dispersion relation (only consider *p*‐polarized wave) reads as follows [27]

(7) $$\begin{array}{c} \eta_{0}\left(\sigma_{x}k_{x}^{2}+\sigma_{y}k_{y}^{2}\right)=2ik_{\rho }k_{0}, \end{array}$$

where vacuum impedance $\eta_{0}=\sqrt{\mu_{0}/\varepsilon_{0}}$. Thus, the dispersion of PhPs in *α*‐MoO_3_ results in strong angular dependence on *φ*, as expected due to the strong in‐plane anisotropies.

Notably, we adopt the first‐order approximation arctan(*x*) = *x* to linearize the dispersion relation in 3D case (Equation (6)) and get the following equation:

(8) $$\begin{array}{c} k_{\rho }=\frac{\rho }{t}\left(2\frac{\varepsilon_{1}\rho }{\varepsilon_{z}}+m\pi \right). \end{array}$$

After performing some algebra and matching coefficients of $k_{x}^{2}$ and $k_{y}^{2}$ terms in Equations (7) and (8), we get the direct relation between ε and σ

(9) $$\begin{array}{c} \sigma_{\alpha }=-i\omega \varepsilon_{0}\varepsilon_{\alpha }t-\omega \varepsilon_{0}\varepsilon_{\alpha }m\pi \sqrt{\frac{\varepsilon_{z}}{\varepsilon_{x}k_{x}^{2}+\varepsilon_{y}k_{y}^{2}}}, \end{array}$$

where α=x,y. The second term on the right of Equation (9) indicates both the feature of higher‐order modes and localization because of the inclusion of *m* and wavevector, $k_{x}$ and $k_{y}$, respectively. The out‐of‐plane dimensional permittivity $\varepsilon_{z}$ occurs in the generalized surface model, suggesting the essential role of out‐of‐plane dimension in correcting the surface model.

**FIGURE 4:**
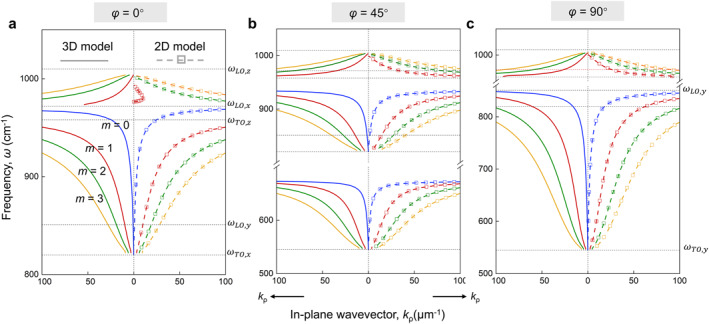
Dispersion of PhPs in an *α*‐MoO_3_ plate with thickness of 200 nm for different value of *φ*: (a) 0°, (b) 45°, (c) 90° calculated by 3D (left) and 2D (right) models. Dotted horizontal lines indicate the LO and TO phonon frequencies along the *x*‐, *y*‐, and *z*‐crystallographic directions of *α*‐MoO_3_, respectively. The blue, red, green, and yellow curves denote the *m* = 0, *m* = 1, *m* = 2, and *m* = 3 modes, respectively.

To verify our generalized 2D method, we investigate the dispersion of PhPs at φ=0°, 45°, and 90° as shown in Figure 4a–c. As expected, the multi‐branched PhPs including fundamental and higher‐order modes can be excited in generalized 2D model. Specifically, in RB 1 and RB 2 spectral regions, both the two models exhibit multiple dispersion curves, representing the excitation of fundamental and higher‐order PhPs, respectively. Note that in the configuration of *α*‐MoO_3_ slab, the fundamental PhPs mode corresponds to surface‐confined hyperbolic polaritons while higher‐order modes represent volume‐confined hyperbolic polaritons for the transition region between RB 2 and RB 3 [27]. These two types of hyperbolic polaritons are transformed into surface modes when the 2D model is used. Furthermore, one can see that the PhPs mode of *m* = 1 adopting generalized 2D model in RB 3 is obviously different from that using 3D model at *φ* = 0°. In comparison, for *φ* = 45° (Figure 4b) and 90° (Figure 4c), the dispersion curves adopting the two models are nearly consistent regardless of spectral regions. Therefore, Figure 4 demonstrates that the second term on the right side of Equation (9) compensates for the higher‐order modes resulting from phases wrapping caused by the dimension in the out‐of‐plane direction.

**FIGURE 5:**
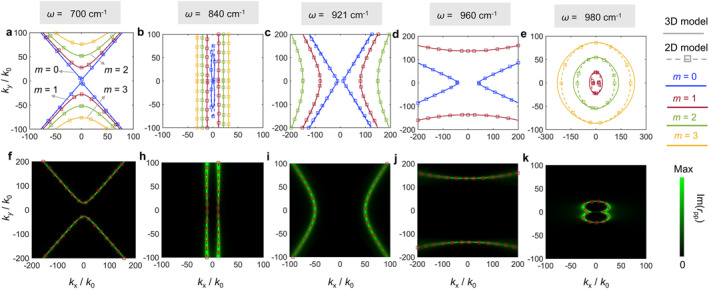
Schematic diagram showing the dispersion and corresponding imaginary part of the Fresnel coefficient, *r*_pp_, of PhPs in *k*_*x*_–*k*_*y*_ momentum space for different frequencies. Top (a–e): the IFCs adopts 3D model (solid curves) and generalized 2D model (dashed curves). The blue, red, green, and yellow curves denote the *m* = 0, *m* = 1, *m* = 2, and *m* = 3 modes, respectively. Bottom (f–k) represent the IFCs (red dashed curve) and Im(*r*_pp_) of the order *m* = 1 mode based on the generalized 2D model. The thickness is 200 nm.

The discernible distinctions between the generalized 2D model and 3D model can be subjected to a more comprehensive and meticulous analysis. To illustrate the origin of distinctions, the in‐plane hyperbolic PhPs are investigated. As is shown in Figure 5, we plot the IFCs in the *k*_x_‐*k*_y_ momentum space at different frequencies. We consider *ω* = 700, 840, 921, 960 and 980 cm^−1^, which represent the typical frequencies in RB 1, the transition region between RB 1 and RB 2, RB 2, the transition region between RB 2 and RB 3, and RB 3, respectively. In Figure 5, we observe that fundamental mode and higher‐order modes exist in the generalized 2D model and are nearly consistent with that in 3D model. Using either 3D model or generalized 2D model, the excited PhPs exhibit the in‐plane hyperbolic features as shown in Figure 5a,c. The color plots in the bottom denote the imaginary part of Fresnel reflection coefficient, Im(*r*_pp_), which agrees excellently with the analytical dispersion curves of order *m* = 1 mode, calculated with the generalized 2D model. This demonstrates the validity of our corrected model, although only the color plot of Im(*r*_pp_) of the *m* = 1 mode is used to compare to the theoretical analysis. At the same time, the difference between two models is obvious in Figure 5b,e, where the non‐overlap of dispersive curves using the two models is evident, especially in the *y* direction in Figure 5b and the *x* direction in Figure 5e. In Ref. [27], we revealed that the relative difference in in‐plane polaritons along the [100] and [001] crystal directions between these two models stems from the assumption in which the first order approximation arctan(x)=x requires that *x* approaches zero (i.e., $\rho /\varepsilon_{z}\to 0$). This explains the inconsistency of the blue curves in the [001] crystal direction as shown in Figure 5b. Similar to the fundamental mode, the inconsistency of dispersion for higher‐order modes as shown in Figure 5e is associated with the magnitude of the value of $\sqrt{\varepsilon_{z}/\left(\varepsilon_{x}\;\cos^{2}\;\varphi +\varepsilon_{y}\;\sin^{2}\;\varphi \right)}$.

To further elucidate the effect of permittivity on the polaritonic difference, we provide a quantitative assessment of the relative difference between the 2D and 3D models in the Supporting Information S1. The results show that the relative difference is inversely proportional to the value of permittivity (see the details in the Supporting Information S1).

### 2.3 Contributions of Different Modes to Enhanced Light–Matter Interactions

The generalized 2D model achieves the separation of different polaritonic modes excited in the vdW slab configuration by isolating the *m* value. Hence, it enables us to investigate light–matter interaction enhancement contributed by each polaritonic mode in the multi‐mode system. Specifically, we study photonic LDOS in a vacuum near an interface. The LDOS is a fundamental quantity, because many macroscopic quantities, such as the near‐field radiative heat transfer between two bodies [49, 50, 51] and Purcell factor, can be derived from it [52, 53, 54]. In the near‐field region, emerging resonant surface wave in polar material prominently boosts the LDOS in vacuum near the interface, thereby breaking Planck's blackbody limit of heat transfer [55, 56, 57, 58]. More photonic tunneling channels can be opened by using the multiple high‐*k* modes in naturally hyperbolic materials such as h‐BN and *α*‐MoO_3_. However, individual contribution of different order polaritonic modes to LDOS remains unexplored, which is essential for interpreting the enhanced radiative heat transfer.

Here, we calculate the LDOS to evaluate the effect of each polaritonic mode on light–matter interactions. According to Ref. [50, 51], the total LDOS is the sum of electric and magnetic contributions. We shall emphasize that in this paper, only the electric LDOS is considered due to the nonmagnetic material of h‐BN and *α*‐MoO_3_. Thus, the expression of electric LDOS (at frequency *ω* and height *z* above the interface in vacuum) reads as follows [50, 51]:

(10) $$\begin{array}{c} \rho \left(z,\omega \right)=\frac{\omega }{\pi c^{2}}\text{ImTr}\left[G^{E}\left(r_{i},r_{j},\omega \right)\right], \end{array}$$

where G^E^ is the electric‐field Green's function, relating the electric field at ***r***_*i*_, which is generated from the source particle at ***r***_*j*_ through the surface reflection [59, 60]. The details are given in Supporting Information S1.

**FIGURE 6:**
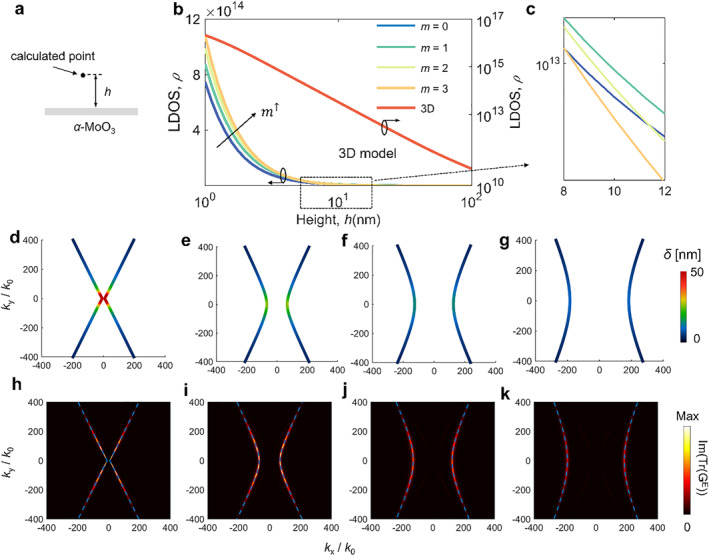
The photonic LDOS above the vdW materials. (a) Schematic of the point in vacuum where LDOS is calculated above an *α*‐MoO_3_ slab. *h* denotes the distance from the vacuum/*α*‐MoO_3_ slab interface. (b) The LDOS contributed from different order polaritonic modes versus the height at the frequency of 900 cm^−1^. (c) The enlargement where the height varies from 8 to 12 nm. (d–g) The attenuation length of corresponding order mode, which is calculated based on δ=1/Imkz0. (h–k) The distribution of imaginary part of the trace of Green's function, Im (Tr(G^E^)) in *k*_*x*_–*k*_*y*_ space for the case of *α*‐MoO_3_ slab at the height of 10 nm. The dashed curves correspond to the analytical dispersion of polaritons of *m* = 0 (h); *m* = 1 (i); *m* = 2 (j); and *m* = 3 (k) modes, respectively. The thickness of *α*‐MoO_3_ is 100 nm.

We calculate the LDOS at the height of *h* above the *α*‐MoO_3_ with 100 nm thickness, schematically illustrated by Figure 6a. The result is shown in Figure 6b at frequency fixed to 900 cm^−1^. The contribution from first four modes, that is, *m* = 0, *m* = 1, *m* = 2, *m* = 3 can be seen directly via the generalized 2D model. One can see that the trend varying with the height for fundamental and higher‐order modes is the same as that based on 3D model (Right of Figure 6b). At small height (about 1 nm), the local density of electromagnetic energy emitted by the *α*‐MoO_3_ plate is dominated by the *m* = 3 mode. With further increase the height, the mode that contributes most to LDOS changes from the *m* = 3 one to *m* = 1, as shown in Figure 6c. When the height is increased to 100 nm, the fundamental mode dominates. The variation with height is due to the vertical confinement of excited modes.

To explain this, we plot the attenuation length of fundamental and higher‐order modes in Figure 6d–g. Note that the contribution is negligible only when the attenuation length of polaritonic mode is smaller than the height of the dipole. The definition of attenuation length is δ=1/Imkz0 [26], which is closely related to excited wavevector. It can be inferred that evanescent waves are easily filtered by the vacuum at larger *k* region (Imkz0>1/h). Comparing Figure 6d with Figure 6g, the maximum attenuation length of fundamental mode exceeds 50 nm while that of *m* = 3 is less than 20 nm, indicating that the contribution of *m* = 3 mode to LDOS at *h* > 20 nm is negligible. However, when the height is 1 nm, the *m* = 3 mode dominates because of the large wavevector integration space. This integration space dependence in turn makes higher‐order modes (e.g., *m* = 3) easier to be filtered at large height. We can infer that the higher‐order mode is more confined on the vacuum/slab interface, leading to stronger contribution to LDOS at smaller height. At the height of 10 nm, one can see that the contribution from the *m* = 1 mode plays a dominant role (see Figure 6c). To get more physical insight, we calculate the trace of reflected Green's function at frequency of 900 cm^−1^ using both the 3D and generalized 2D models, as shown in Figure 6h–k. The bright hyperbolic stripe in *k*_*x*_–*k*_*y*_ space indicates that hyperbolic polaritons significantly enhance the LDOS for all modes. We find the higher order modes occupy higher momentum spaces. Specifically, the *k*_*ρ*_ is less than 200 *k*_0_ for *m* = 0 mode while it is extended to nearly 400 *k*_0_ for *m* = 3 mode.

Besides, we calculate the LDOS above the h‐BN slab as shown in Supporting Information S1: Section S7. One can see that the trend varying with the height for fundamental and higher‐order modes is the same with that in the case of *α*‐MoO_3_. For h‐BN, the IFCs of phonon polaritons are circular due to in‐plane isotropy, which also enhances the LDOS, as shown in Supporting Information S1: Figure S7f–i.

Finally, we discuss the validity of the synthesized GSCM. We expect that by using this model theory, we will be able to obtain results that are consistent with those obtained from the full 3D model. Since there are multiple waveguide modes in the thin slab with finite thickness, increasing the participating numbers of polaritonic modes can improve accuracy when using synthesized GSCM. However, not all modes contribute to the near‐field light–matter interaction. In Supporting Information S1: Section S3, we directly compare the synthesized GSCM and full 3D model in terms of reflection coefficient and LDOS. The results show that when the number exceeds 9, the relative difference between the two models decreases to below 0.05, thereby proving the validity of the GSCM. Interestingly, by investigating the dependence of the validity on the thickness, we demonstrate that the thickness of the slab is not associated with the accuracy of the synthesized GSCM (Supporting Information S1: Section S4). This is because the assumptions mentioned above have nothing to do with the thickness.

## 3 Conclusions

Previous 2D model only considers the fundamental mode of multi‐branched phonon polaritons, neglecting the out‐of‐plane dimension. We develop a generalized 2D surface conductivity model, incorporating higher‐order modes, to bridge this gap, in anisotropic vdW materials. We comprehensively compare the dispersion relation in biaxial *α*‐MoO_3_ plate using both 3D model and our corrected 2D model. By considering the permittivity in the out‐of‐plane dimension, the multi‐branched hyperbolic polaritons excited in the 3D model also appear in the 2D model and are in good agreement with the imaginary part of Fresnel reflection coefficient.

In addition, the generalized 2D model can separate the multi‐branched hyperbolic polaritons, allowing the investigation of the individual polaritonic mode. We reveal that the contribution of each mode to the LDOS in vacuum above the vdW *α*‐MoO_3_ and h‐BN plates. The trend of LDOS with height is mediated by the vertical confinement length, that is, attenuation length. We find that the dominant mode for enhancing LDOS shows a strong position dependence, requiring its vacuum distance less than the confinement length of the polaritonic mode. We demonstrate that higher‐order modes have small attenuation length owing to large excited wavevector space. Thus, the higher‐order modes dominate the LDOS for small vacuum height, while the fundamental mode contributes the most as the distance increases.

Although *α*‐MoO_3_ is chosen for demonstration in this paper, the conclusions obtained can be extended to other vdW materials, such as *α*‐V_2_O_5_ [61] and many others. Therefore, our findings promise new freedoms of the dispersion geometry design for tailored applications, like the in‐plane modulation of light flow at the nanoscale. Furthermore, this study provides fundamentally relevant insights into understanding in‐plane anisotropic polaritons of vdW materials.

## 4 Methods

### 4.1 Numerical Calculations

LDOS calculation is carried out by numerical evaluating the integral in Equation (10). The Fresnel reflection coefficient which is used in Im(*r*_pp_), and LDOS calculation are based on the Fresnel formulas and Transfer Matrix Method for the in‐plane isotropic and anisotropic materials, respectively (see Supporting Information S1 for details). For anisotropic surface sheet with the diagonal conductivity tensor, diag $\left(\begin{array}{cc} \sigma_{x} & \sigma_{y} \end{array}\right)$, in which $\sigma_{x\left(y\right)}$ is given by Equation (9), the boundary conditions change from the continuous in‐plane magnetic field to differential current density at upper (1) and lower (2) interfaces [36]:

(11) $$\begin{array}{c} \left(\begin{array}{c} h_{1,x}\\ h_{1,y} \end{array}\right)-\left(\begin{array}{c} h_{2,x}\\ h_{2,y} \end{array}\right)=\left(\begin{array}{c} J_{x}\\ J_{y} \end{array}\right), \end{array}$$

where *h*_*x*(*y*)_ represents the in‐plane magnetic field component along *x*‐(*y*‐)axis direction and *J*_*x*(*y*)_ is the current density. The current density is associated with the continuous in‐plane electric field, *e*_*x*,*y*_, given by the following equation:

(12) $$\begin{array}{c} \left(\begin{array}{c} J_{x}\\ J_{y} \end{array}\right)=\left(\begin{array}{cc} \sigma_{x} & 0\\ 0 & \sigma_{y} \end{array}\right)\left(\begin{array}{c} e_{x}\\ e_{y} \end{array}\right). \end{array}$$

### 4.2 Full‐Wave Simulations

The full‐wave numerical simulations are performed to calculate the real part of the vertical component of the near‐field Re(*E*_*z*_(*x*,*y*)), that is, Figures 1 and 3, using the code based on Green's function method [52]. The PhPs are launched by vertically oriented electric point dipole sources placed 50 nm above h‐BN or *α*‐MoO_3_ uppermost surface. We record the electric field distribution at 20 nm on the top of the surface. 3D model and 2D model strictly treat the slab as a surface without thickness and 3D slab with finite thickness, respectively. See Section S8 in Supporting Information S1 for the details of Green's function method. The distributions of electric field of PhPs supported by in‐plane hyperbolic *α*‐MoO_3_ slab with the thickness of 100 and 150 nm are calculated in Supporting Information S1: Section S9.

## Author Contributions

**Shuo Chen:** conceptualization, investigation, visualization, writing – original draft, writing – review and editing. **Yuchen Sun:** conceptualization, investigation, validation. **Jing Wu:** methodology, validation. **Ceji Fu:** writing – review and editing, investigation, funding acquisition. **Guangwei Hu:** conceptualization, writing – review and editing, validation, methodology, funding acquisition, investigation.

## Funding

This work was supported by the China Scholarship Council (No. 202306010144), the National Natural Science Foundation of China (No. 51576004). G. H. acknowledges the Nanyang Assistant Professorship Start‐up Grant, National Research Foundation of Singapore through the Competitive Research Program (NRF‐CRP29‐2022‐0003) and Ministry of Education (Singapore) under Grant AcRF TIER 1 (No. RG61/23) and AcRF TIER2 (MOE‐T2EP50224‐0044). J. W. acknowledges support from the National Natural Science Foundation of China (Grant No. 62404042) and the Center for Fundamental and Interdisciplinary Sciences of Southeast University.

## Conflicts of Interest

The authors declare no conflicts of interest.

## Supporting information


Supporting Information S1

## Data Availability

The data that support the findings of this study are available from the corresponding author upon reasonable request.

## References

[1] Q. Zhang , G. Hu , W. Ma , et al., “Interface Nano‐Optics With van der Waals Polaritons,” Nature 597, no. 7875 (2021): 187–195, 10.1038/s41586-021-03581-5. 34497390

[2] K. Chaudhary , M. Tamagnone , M. Rezaee , et al., “Engineering Phonon Polaritons in van der Waals Heterostructures to Enhance In‐Plane Optical Anisotropy,” Science Advances 5, no. 4 (2019): eaau7171, 10.1126/sciadv.aau7171. 30993198 PMC6461454

[3] E. Galiffi , G. Carini , X. Ni , et al., “Extreme Light Confinement and Control in Low‐Symmetry Phonon‐Polaritonic Crystals,” Nature Review Materials 9, no. 1 (2024): 9–28, 10.1038/s41578-024-00674-3.

[4] N. Rivera and I. Kaminer , “Light–Matter Interactions With Photonic Quasiparticles,” Nature Reviews Physics 2, no. 10 (2020): 538–561, 10.1038/s42254-020-0224-2.

[5] D. Lee , S. So , G. Hu , et al., “Hyperbolic Metamaterials: Fusing Artificial Structures to Natural 2D Materials,” eLight 2, no. 1 (2022): 1–23, 10.1186/s43593-021-00008-6.

[6] P. Li , M. Lewin , A. V. Kretinin , et al., “Hyperbolic Phonon‐Polaritons in Boron Nitride for Near‐Field Optical Imaging and Focusing,” Nature Communications 6, no. 1 (2015): 7507, 10.1038/ncomms8507. PMC4491815 26112474

[7] Z. Zheng , N. Xu , S. L. Oscurato , et al., “A Mid‐Infrared Biaxial Hyperbolic van der Waals Crystal,” Science Advances 5, no. 5 (2019): eaav8690, 10.1126/sciadv.aav8690. 31139747 PMC6534390

[8] Z. Dai , G. Hu , G. Si , et al., “Edge‐Oriented and Steerable Hyperbolic Polaritons in Anisotropic van der Waals Nanocavities,” Nature Communications 11, no. 1 (2020): 6086, 10.1038/s41467-020-19913-4. PMC7705012 33257664

[9] L. Liu , L. Xiong , C. Wang , et al., “Long‐Range Hyperbolic Polaritons on a Non‐Hyperbolic Crystal Surface,” Nature 644, no. 8075 (2025): 76–82, 10.1038/s41586-025-09288-1. 40670785

[10] A. I. F. Tresguerres‐Mata , C. Lanza , J. Taboada‐Gutiérrez , et al., “Observation of Naturally Canalized Phonon Polaritons in LiV_2_O_5_ Thin Layers,” Nature Communications 15, no. 1 (2024): 2696, 10.1038/s41467-024-46935-z. PMC10973474 38538588

[11] B. Zhao , B. Guizal , Z. M. Zhang , S. Fan , and M. Antezza , “Near‐Field Heat Transfer Between Graphene/hBN Multilayers,” Physical Review B: Condensed Matter 95, no. 24 (2017): 245437, 10.1103/PhysRevB.95.245437.

[12] K. Shi , F. Bao , and S. He , “Enhanced Near‐Field Thermal Radiation Based on Multilayer Graphene‐hBN Heterostructures,” ACS Photonics 4, no. 4 (2017): 971–978, 10.1021/acsphotonics.7b00037.

[13] C. Zhou , Y. Zhang , and H. Yi , “Enhancement and Manipulation of Near‐Field Thermal Radiation Using Hybrid Hyperbolic Polaritons,” Langmuir 38, no. 25 (2022): 7689–7698, 10.1021/acs.langmuir.2c00467. 35699142

[14] J. Zhang , B. Yang , K. Shi , H. Liu , and X. Wu , “Polariton Hybridization Phenomena on Near‐Field Radiative Heat Transfer in Periodic Graphene/α‐MoO_3_ Cells,” Nanophotonics 12, no. 10 (2023): 1833–1846, 10.1515/nanoph-2022-0730. 39635143 PMC11502109

[15] A. Bylinkin , M. Schnell , M. Autore , et al., “Real‐Space Observation of Vibrational Strong Coupling Between Propagating Phonon Polaritons and Organic Molecules,” Nature Photonics 15, no. 3 (2021): 197–202, 10.1038/s41566-020-00725-3.

[16] A. Bylinkin , F. Calavalle , M. Barra‐Burillo , et al., “Dual‐Band Coupling of Phonon and Surface Plasmon Polaritons With Vibrational and Electronic Excitations in Molecules,” Nano Letters 23, no. 9 (2023): 3985–3993, 10.1021/acs.nanolett.3c00768. 37116103

[17] A. Bylinkin , S. Castilla , T. M. Slipchenko , et al., “On‐Chip Phonon‐Enhanced IR Near‐Field Detection of Molecular Vibrations,” Nature Communications 15, no. 1 (2024): 8907, 10.1038/s41467-024-53182-9. PMC11484778 39414807

[18] X. Lin , Yi Yang , N. Rivera , et al., “All‐Angle Negative Refraction of Highly Squeezed Plasmon and Phonon Polaritons in Graphene–Boron Nitride Heterostructures,” Proceedings of the National Academy of Sciences 114, no. 26 (2017): 6717–6721, 10.1073/pnas.1701830114. PMC5495251 28611222

[19] A. J. Sternbach , S. L. Moore , A. Rikhter , et al., “Negative Refraction in Hyperbolic Hetero‐Bicrystals,” Science 379, no. 6632 (2023): 555–557, 10.1126/science.adf1065. 36758086

[20] P. Li , I. Dolado , F. J. Alfaro‐Mozaz , et al., “Infrared Hyperbolic Metasurface Based on Nanostructured van der Waals Materials,” Science 359, no. 6378 (2018): 892–896, 10.1126/science.aaq1704. 29472478

[21] M. He , T. G. Folland , J. Duan , et al., “Anisotropy and Modal Hybridization in Infrared Nanophotonics Using Low‐Symmetry Materials,” ACS Photonics 9, no. 4 (2022): 1078–1095, 10.1021/acsphotonics.1c01486.

[22] Y. Bai , Q. Zhang , T. Zhang , et al., “Airy‐Like Hyperbolic Shear Polaritons in High Symmetry van der Waals Crystals,” Laser & Photonics Reviews 18, no. 7 (2024): 2400041, 10.1002/lpor.202400041.

[23] W. Ma , P. Alonso‐González , S. Li , et al., “In‐Plane Anisotropic and ultra‐low‐loss Polaritons in a Natural van der Waals Crystal,” Nature 562, no. 7728 (2018): 557–562, 10.1038/s41586-018-0618-9. 30356185

[24] G. Hu , A. Krasnok , Y. Mazor , C. W. Qiu , and A. Alù , “Moiré Hyperbolic Metasurfaces,” Nano Letters 20, no. 5 (2020): 3217–3224, 10.1021/acs.nanolett.9b05319. 32298129

[25] G. Hu , Q. Ou , G. Si , et al., “Topological Polaritons and Photonic Magic Angles in Twisted α‐MoO_3_ Bilayers,” Nature 582, no. 7811 (2020): 209–213, 10.1038/s41586-020-2359-9. 32528096

[26] P. Li , G. Hu , I. Dolado , et al., “Collective Near‐Field Coupling and Nonlocal Phenomena in Infrared‐Phononic Metasurfaces for Nano‐Light Canalization,” Nature Communications 11, no. 1 (2020): 3663, 10.1038/s41467-020-17425-9. PMC7374561 32694591

[27] S. Chen , X. Wu , and C. Fu , “Comparative Analysis of Two Models for Phonon Polaritons in van der Waals Materials: 2D and 3D,” Nanoscale 15, no. 44 (2023): 17889–17898, 10.1039/D3NR03879C. 37889109

[28] S. Chen , H. Liu , B. Wu , X. Wu , and C. Fu , “Comparative Analysis of Two Models for the Optical Response of a Thin Slab: A Film of Finite Thickness Versus a Surface Current Sheet,” Physical Chemistry Chemical Physics 25, no. 8 (2023): 6194–6202, 10.1039/D2CP05282B. 36752694

[29] J. D. Caldwell , I. Aharonovich , G. Cassabois , J. H. Edgar , B. Gil , and D. N. Basov , “Photonics With Hexagonal Boron Nitride,” Nature Review Materials 4, no. 8 (2019): 552–567, 10.1038/s41578-019-0124-1.

[30] A. Kumar , T. Low , K. H. Fung , P. Avouris , and N. X. Fang , “Tunable Light–Matter Interaction and the Role of Hyperbolicity in Graphene–hBN System,” Nano Letters 15, no. 5 (2015): 3172–3180, 10.1021/acs.nanolett.5b01191. 25897983

[31] G. Hu , C. Zheng , J. Ni , C.‐W. Qiu , and A. Alù , “Enhanced Light‐Matter Interactions at Photonic Magic‐Angle Topological Transitions,” Applied Physics Letters 118, no. 21 (2021): 211101, 10.1063/5.0052580.

[32] C. Zheng , G. Hu , X. Liu , X. Kong , L. Wang , and C.‐W. Qiu , “Molding Broadband Dispersion in Twisted Trilayer Hyperbolic Polaritonic Surfaces,” ACS Nano 16, no. 8 (2022): 13241–13250, 10.1021/acsnano.2c07123. 35938977

[33] V. W. Brar , M. S. Jang , M. Sherrott , et al., “Hybrid Surface‐Phonon‐Plasmon Polariton Modes in Graphene/Monolayer h‐BN Heterostructures,” Nano Letters 14, no. 7 (2014): 3876–3880, 10.1021/nl501096s. 24874205

[34] M. Esslinger , R. Vogelgesang , N. Talebi , et al., “Tetradymites as Natural Hyperbolic Materials for the Near‐Infrared to Visible,” ACS Photonics 1, no. 12 (2014): 1285–1289, 10.1021/ph500296e.

[35] N. Talebi , C. Ozsoy‐Keskinbora , H. M. Benia , K. Kern , C. T. Koch , and P. A. van Aken , “Wedge Dyakonov Waves and Dyakonov Plasmons in Topological Insulator Bi_2_Se_3_ Probed by Electron Beams,” ACS Nano 10, no. 7 (2016): 6988–6994, 10.1021/acsnano.6b02968. 27309040

[36] H. Wu , X. Liu , Y. Cai , L. Cui , and Y. Huang , “Near‐Field Radiative Heat Transfer Modulated by Nontrivial Topological Surface States,” Materials Today Physics 27 (2022): 100825, 10.1016/j.mtphys.2022.100825.

[37] A. J. Sternbach , S. H. Chae , S. Latini , et al., “Programmable Hyperbolic Polaritons in van der Waals Semiconductors,” Science 371, no. 6529 (2021): 617–620, 10.1126/science.abe9163. 33542134

[38] F. H. Feres , R. A. Mayer , L. Wehmeier , et al., “Sub‐Diffractional Cavity Modes of Terahertz Hyperbolic Phonon Polaritons in Tin Oxide,” Nature Communications 12, no. 1 (2021): 1995, 10.1038/s41467-021-22209-w. PMC8012705 33790286

[39] P. Yeh , Optical Waves in Layered Media (Wiley, 1988).

[40] Z. Zhang , Nano/Microscale Heat Transfer (McGraw‐Hill, 2007).

[41] O. Y. Yermakov , A. I. Ovcharenko , M. Song , A. A. Bogdanov , I. V. Iorsh , and Yu. S. Kivshar , “Hybrid Waves Localized at Hyperbolic Metasurfaces,” Physical Review B: Condensed Matter 91, no. 23 (2015): 235423, 10.1103/PhysRevB.91.235423.

[42] J. S. Gomez‐Diaz , M. Tymchenko , and A. Alù , “Hyperbolic Plasmons and Topological Transitions Over Uniaxial Metasurfaces,” Physical Review Letters 114, no. 23 (2015): 233901, 10.1103/PhysRevLett.114.233901. 26196803

[43] G. Álvarez‐Pérez , T. G. Folland , I. Errea , et al., “Infrared Permittivity of the Biaxial van der Waals Semiconductor α‐MoO_3_ From Near‐ and Far‐Field Correlative Studies,” Advanced Materials 32, no. 29 (2020): 1908176, 10.1002/adma.201908176. 32495483

[44] G. Álvarez‐Pérez , K. V. Voronin , V. S. Volkov , P. Alonso‐González , and A. Y. Nikitin , “Analytical Approximations for the Dispersion of Electromagnetic Modes in Slabs of Biaxial Crystals,” Physical Review B: Condensed Matter 100, no. 23 (2019): 235408, 10.1103/PhysRevB.100.235408.

[45] Y. Zeng , Q. Ou , Lu Liu , et al., “Tailoring Topological Transitions of Anisotropic Polaritons by Interface Engineering in Biaxial Crystals,” Nano Letters 22, no. 10 (2022): 4260–4268, 10.1021/acs.nanolett.2c00399. 35442697

[46] S. J. Yu , H. Yao , G. Hu , et al., “Hyperbolic Polaritonic Rulers Based on van der Waals α‐MoO_3_ Waveguides and Resonators,” ACS Nano 17, no. 22 (2023): 23057–23064, 10.1021/acsnano.3c08735. 37948673

[47] L. Wehmeier , S.‐J. Yu , X. Chen , et al., “Tunable Phonon Polariton Hybridization in a van der Waals Hetero‐Bicrystal,” Advanced Materials 36, no. 33 (2024): 2401349, 10.1002/adma.202401349. 38657644

[48] S. Chen , X. Wu , and C. Fu , “Active Tuning of Anisotropic Phonon Polaritons in Natural van der Waals Crystals With Negative Permittivity Substrates and Its Application in Energy Transport,” Opto‐Electronic Science 3, no. 5 (2024): 240002, 10.29026/oes.2024.240002.

[49] J. P. Mulet , K. Joulain , R. Carminati , and J.‐J. Greffet , “Enhanced Radiative Heat Transfer at Nanometric Distances,” Microscale Thermophysical Engineering 6, no. 3 (2002): 209–222, 10.1080/10893950290053321.

[50] R. Carminati and J. J. Greffet , “Near‐Field Effects in Spatial Coherence of Thermal Sources,” Physical Review Letters 82, no. 8 (1999): 1660–1663, 10.1103/PhysRevLett.82.1660.

[51] K. Joulain , R. Carminati , J.‐P. Mulet , and J.‐J. Greffet , “Definition and Measurement of the Local Density of Electromagnetic States Close to an Interface,” Physical Review B: Condensed Matter 68, no. 24 (2003): 245405, 10.1103/PhysRevB.68.245405.

[52] L. Novotny and B. Hecht , Principles of Nano‐Optics, 2nd ed. (Cambridge Univ. Press, 2012).

[53] M. Fox , Optical Properties of Solids, 2nd ed. (Oxford Univ. Press, 2010).

[54] A. Losquin and M. Kociak , “Link Between Cathodoluminescence and Electron Energy Loss Spectroscopy and the Radiative and Full Electromagnetic Local Density of States,” ACS Photonics 2, no. 11 (2015): 1619–1627, 10.1021/acsphotonics.5b00416.

[55] J. DeSutter , L. Tang , and M. Francoeur , “A near‐field Radiative Heat Transfer Device,” Nature Nanotechnology 14, no. 8 (2019): 751–755, 10.1038/s41565-019-0483-1. 31263192

[56] A. Fiorino , D. Thompson , L. Zhu , et al., “A Thermal Diode Based on Nanoscale Thermal Radiation,” ACS Nano 12, no. 6 (2018): 5774–5779, 10.1021/acsnano.8b01645. 29790344

[57] P. Ben‐Abdallah and S. A. Biehs , “Near‐Field Thermal Transistor,” Physical Review Letters 112, no. 4 (2014): 044301, 10.1103/PhysRevLett.112.044301. 24580455

[58] N. Iqbal , S. Zhang , S. Wang , et al., “Measuring Near‐Field Radiative Heat Transfer in a Graphene‐SiC Heterostructure,” Physical Review Applied 19, no. 2 (2023): 024019, 10.1103/PhysRevApplied.19.024019.

[59] S. Chen , C. Fu , and G. Hu , “Phonon‐Polariton‐Mediated Configurable Radiative Thermal Router,” ACS Photonics 12, no. 1 (2024): 271–281, 10.1021/acsphotonics.4c01622.

[60] S. Chen , X. Wang , C. Fu , and G. Hu , “Analyzing and Quantifying Symmetry Breaking of Anisotropic Shear Polaritons in Monoclinic Crystal Slabs,” Nanoscale 17, no. 38 (2025): 22218–22225, 10.1039/D5NR02806J. 40947962

[61] J. Taboada‐Gutiérrez , G. Álvarez‐Pérez , J. Duan , et al., “Broad Spectral Tuning of Ultra‐Low‐Loss Polaritons in a van der Waals Crystal by Intercalation,” Nature Materials 19, no. 9 (2020): 964–968, 10.1038/s41563-020-0665-0. 32284598

