# Supplementary material for: Generalized Surface Conductivity Model for Anisotropic Phonon Polaritons in van der Waals Slabs
Source: Nanophotonics. 2026 Jan 20;15(2):e70010. doi: 10.1002/nap2.70010 (PMC12964996; doi:10.1002/nap2.70010)
Supplement: Supplementary file 1 — Supporting Information S1 [file NAP2-15-e70010-s001.pdf]

Supplementary Material for:

Generalized surface conductivity model for anisotropic phonon polaritons in van der Waals slabs

Shuo Chen[1,2,3], Yuchen Sun[2], Jing Wu[4], Ceji Fu[1,*], and Guangwei Hu[2,3*]

[1]LTCS, School of Mechanics and Engineering Science, Peking University, Beijing 100871, P. R. China

[2]School of Electrical and Electronic Engineering, Nanyang Technological University, Singapore 639798, Singapore

[3]CINTRA (CNRS–International-NTU-THALES-Research Alliances/UMI 3288), 50 Nanyang Drive, Singapore, 637553 Singapore

[4]School of Electronic Science and Engineering, Southeast University, Nanjing 210096, P. R. China

*Corresponding author: guangwei.hu@ntu.edu.sg; cjfu@pku.edu.cn

Table of Contents

Section 1. The reflection matrix of the anisotropic slabs covered with anisotropic surface materials

Section 2. The dielectric function of h-BN and α-MoO_3

Section 3. The validity of the synthesized generalized surface conductivity model

Section 4. The effect of thickness of the slab on the validity of GSCM

Section 5. The analysis of the relative difference between 2D and 3D models in dispersion

Section 6. The electric-field Green's function

Section 7. The photonics LDOS above an h-BN slab

Section 8. The calculation of electric field launched by a z-polarized electric dipole

Section 9. The distribution of electric fields for different thicknesses using GSCM

Section 10. The detailed derivation of Eq. 5

Section 1. The reflection matrix of anisotropic slab covered with anisotropic surface materials

(1) Reflection coefficient of h-BN slab and corresponding surface sheet

For in-plane isotropic plate, i.e., h-BN, the reflection coefficient can be calculated by Fresnel formulas. The corresponding surface conductivity of h-BN slab is also in-plane isotropic. For the suspending h-BN slab (Fig. S1(a)), the reflection coefficients of TM and TE waves take the following forms [1-4]:

$$r_p = \frac{r_{12,p} + r_{23,p} \exp\left(2ik_{2,z}^{p} d\right)}{1 - r_{21,p} r_{23,p} \exp\left(2ik_{2,z}^{p} d\right)}, \tag{S1}$$

and

$$r_s = \frac{r_{12,s} + r_{23,s} \exp\left(2ik_{2,z}^{s} d\right)}{1 - r_{21,s} r_{23,s} \exp\left(2ik_{2,z}^{s} d\right)}, \tag{S2}$$

where 1, 2, and 3 are the indexes for the vacuum region above h-BN film, h-BN film region and the vacuum region below h-BN film region, respectively.

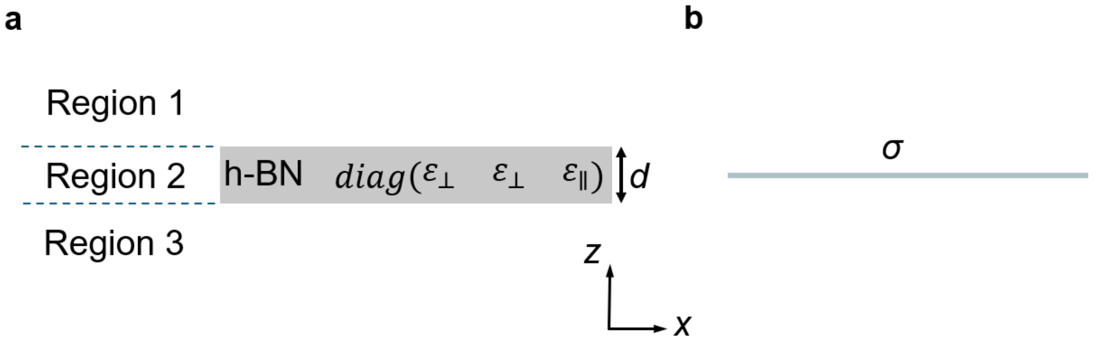

Fig. S1. Schematic of (a) anisotropic h-BN slab and (b) corresponding 2D surface sheet. The optical axis of h-BN slab is parallel to *z* axis and the thickness is *d*. σ denotes the surface conductivity of the surface and the permittivity of the substrate, respectively.

$r_{ab,p(s)}$ denotes the Fresnel reflection coefficient of *p(s)*-polarized waves incident from medium *a* to medium *b*. For the general case where medium *a* and *b* are both anisotropic with permittivity of $diag(\varepsilon_\perp \quad \varepsilon_\perp \quad \varepsilon_\parallel)$ and a 2D surface with conductivity σ is clad between them [2-4],

$$r_{ab,p}=\frac{\varepsilon_{\perp,b}k^p_{a,z}-\varepsilon_{\perp,a}k^p_{b,z}+k^p_{a,z}k^p_{b,z}\dfrac{\sigma}{\omega\varepsilon_0}}{\varepsilon_{\perp,b}k^p_{a,z}+\varepsilon_{\perp,a}k^p_{b,z}+k^p_{a,z}k^p_{b,z}\dfrac{\sigma}{\omega\varepsilon_0}}, \tag{S3}$$

and

$$r_{ab,s}=\frac{k^s_{a,z}-k^s_{b,z}-\sigma\omega\mu_0}{k^s_{a,z}+k^s_{b,z}+\sigma\omega\mu_0}. \tag{S4}$$

Here ε_0 and μ_0 are the vacuum permittivity and permeability, respectively. $k^p_{a(b),z}$ and $k^s_{a(b),z}$ are the normal components of wave vector in medium $a(b)$ for p- or s-polarization, respectively, and are written as [1-4]

$$k^p_{a(b),z}=\sqrt{\varepsilon_{\perp,a(b)}k_0^2-\frac{\varepsilon_{\perp,a(b)}}{\varepsilon_{\parallel,a(b)}}k_\rho^2}, \tag{S5}$$

and

$$k^s_{a(b),z}=\sqrt{\varepsilon_{\perp,a(b)}k_0^2-k_\rho^2}, \tag{S6}$$

where k_ρ is the in-plane wave vector component.

For the structure of Fig. S1(a), superstrate 1 and substrate 3 are both vacuum, so that the Eq. (S5) and Eq. (S6) are simplified to [2-4]

$$k^{p(s)}_{1(3),z}=\sqrt{k_0^2-k_\rho^2}, \tag{S7}$$

We can get the reflection coefficient of monolayer h-BN without 2D surface material by $\sigma = 0$. In addition, for the case of individual 2D surface (Fig. S1(b)), we use the Eq. (S3) and Eq. (S4) to obtain the reflection coefficient.

Note that when a p-polarized wave is incident on hBN interface, the reflected wave is also a p-polarized wave, namely, there is no polarization transformation from the p wave to the s wave.

(2) Reflection coefficient of α-MoO_3 slab and corresponding surface sheet

For in-plane anisotropic plate structure, Fresnel formulas cannot provide analytical expression. Hence, the Transfer Matrix Method (TMM) can be adopted to obtain the reflection matrix [5-7]. The derivation can be seen in Ref. S7. In our calculation, the

anisotropic 2D surface materials are suspended in vacuum. Therefore, it is essential to extend TMM so that the reflection coefficient and transmission coefficient of 2D anisotropic surface materials can be calculated.

For the sake of simplicity, we take monolayer anisotropic plate as an example to derive the reflection matrix, as shown in Fig. S2(a). Note that there are two angles in our calculation, azimuth angle Φ and polarizing angle ψ. The former, azimuth angle Φ, denotes the angle between the plane of incidence and *x*-*z* plane as shown in Fig. S2(a). When Φ is not equal to zero, the plane of incidence is tilled off the *x* axis by an angle Φ. The latter, polarizing angle ψ, indicates the angle between the direction of polarization of the electric field and the plane of incidence. Consequently, $\psi = 0$ and $\frac{\pi}{2}$ corresponds to the transverse magnetic wave and transverse electric wave, respectively.

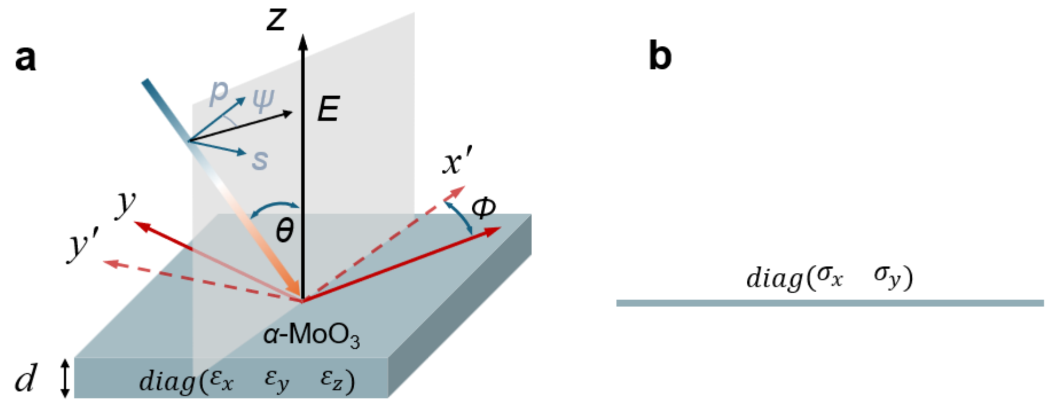

Fig. S2. (a) Schematic of the monolayer anisotropic film and the coordinate systems in this work. The plane of incidence is tilted off the *x*-axis by angle Φ. The direction of polarization of the electric field is tilted off the plane of incidence by angle ψ. The $x'z$ plane coincides with the plane of incidence. (b) Corresponding anisotropic 2D surface material. The anisotropic slab with permittivity tensor of $\bar{\bar{\varepsilon}}$ has a thickness of d. 2D surface sheet has a conductivity tensor of $\bar{\bar{\sigma}}$.

For the anisotropic surface sheet as shown in Fig. S2(b), we can use the extended TMM. In comparison, the boundary condition changes due to the emergence of a surface sheet. When α-MoO_3slab is considered as the surface sheet, the corresponding generalized surface conductivity is anisotropic due to the in-plane anisotropy, which can be expressed as

$$\bar{\bar{\sigma}} = \begin{pmatrix} \sigma_{xx} & \sigma_{xy} \\ \sigma_{yx} & \sigma_{yy} \end{pmatrix}, \tag{S8}$$

The boundary conditions change from the continuous in-plane magnetic field to differential current density at upper (1) and lower (2) interfaces [8]:

$$\begin{pmatrix} H_{1,x} \\ H_{1,y} \end{pmatrix} - \begin{pmatrix} H_{2,x} \\ H_{2,y} \end{pmatrix} = \begin{pmatrix} J_x \\ J_y \end{pmatrix}, \tag{S9}$$

where $H_{x(y)}$ represents the in-plane magnetic field component along x-(y-)axis direction and $J_{x(y)}$ is the current density. The current density is associated with the continuous in-plane electric field, $E_{x,y}$, given by

$$\begin{pmatrix} J_x \\ J_y \end{pmatrix} = \begin{pmatrix} \sigma_{xx} & \sigma_{xy} \\ \sigma_{yx} & \sigma_{yy} \end{pmatrix} \begin{pmatrix} E_x \\ E_y \end{pmatrix}. \tag{S10}$$

Section 2: The dielectric function of h-BN and α-MoO$_3$

The permittivity tensor components of α-MoO_3 can be described by a Lorentz model:

$$\varepsilon_j = \varepsilon_\infty^j \left(1 + \frac{\omega_{L,j}^2 - \omega_{T,j}^2}{\omega_{T,j}^2 - \omega^2 - i\omega\Gamma_j} \right), \tag{S11}$$

where $j = x, y, z$ and the detailed parameters are listed in Table S1 [9]

Table S1. Permittivity values of α-MoO_3

[100] crystalline direction		[001] crystalline direction		[010] crystalline direction	
$\varepsilon_\infty^x(cm^{-1})$	4	$\varepsilon_\infty^y(cm^{-1})$	5.2	$\varepsilon_\infty^z(cm^{-1})$	2.4
$\omega_{T,x}(cm^{-1})$	820	$\omega_{T,y}(cm^{-1})$	545	$\omega_{T,z}(cm^{-1})$	958
$\omega_{L,x}(cm^{-1})$	972	$\omega_{L,y}(cm^{-1})$	851	$\omega_{L,z}(cm^{-1})$	1010
$\Gamma_x(cm^{-1})$	4	$\Gamma_y(cm^{-1})$	4	$\Gamma_z(cm^{-1})$	4

The optical properties of h-BN can be described by Lorentz model [10], whose permittivity is given by

$$\varepsilon_j = \varepsilon_{\infty,j} \left(1 + \frac{\omega_{LO,j}^2 - \omega_{TO,j}^2}{\omega_{TO,j}^2 - \omega^2 - i\omega\Gamma_m} \right), \tag{S12}$$

where $j = \perp, \parallel$ corresponds to the direction perpendicular or parallel to the optical axis. $\omega_{TO,\parallel} = 780cm^{-1}, \omega_{LO,\parallel} = 830cm^{-1}, \Gamma_\parallel = 4cm^{-1}, \varepsilon_{\infty,\parallel} = 2.95$ lead to the lower Reststrahlen band, where $Re(\varepsilon_\parallel) < 0, Re(\varepsilon_\perp) > 0$; $\omega_{TO,\perp} = 1370cm^{-1}, \omega_{LO,\perp} = 1610cm^{-1}, \Gamma_\perp = 5cm^{-1}, \varepsilon_{\infty,\perp} = 4.87$ result in the upper Reststrahlen band where $Re(\varepsilon_\parallel) > 0, Re(\varepsilon_\perp) < 0$.

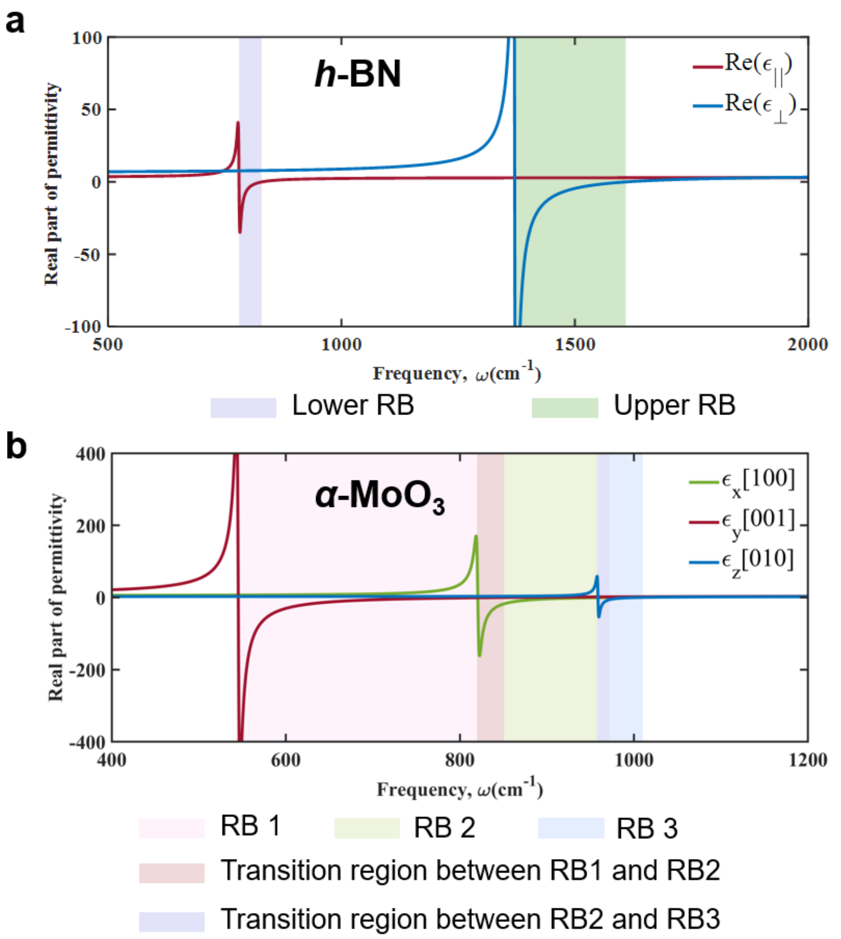

Fig. S3 The real part of permittivity of (a) h-BN and (b) α-MoO_3. The Restrahlen bands are denoted by different color shadows.

Section 3: The validity of the synthesized generalized surface conductivity model

The generalized surface conductivity model (GSCM) provides individual surface conductivity for each polaritonic mode. Thus, the validity of the synthesized GSCM model including all PhPs modes and full 3D strongly depends on the accuracy of each mode. Thus, in this section, we first compare the distribution of Im(r_{pp}) of multiple waveguide modes that are calculated using GSCM and 3D model respectively. There is no doubt that there are certain differences between the results of the 2D model and those of the 3D model. However, as the number of polaritonic modes involved in the calculation increases, the results become closer to those of the 3D full model. Then, we set the LDOS as an example to demonstrate the validity of the synthesized GSCM. We

calculate and compare the enhancement of LDOS contributed from all polaritonic modes using the synthesized GSCM and full 3D model, respectively.

To further demonstrate the validity of the proposed corrected 2D model, we compare the distributions of Im(r_{pp}) in frequency and wavevector space using the synthesized model with those based on the 3D model, as shown in Fig. S4. Three azimuthal angles are chosen: $\varphi = 0^{\circ}, 45^{\circ}$ and 90°. One can see that the bright stripes agree well with analytical dispersion curves for the synthesized model. Multi-branches bright stripes in 2D and 3D model are nearly symmetric with respect to the white line regardless of RB 3 spectral region at $\varphi = 0^{\circ}$. Here, the highest order mode in the synthesized model is $m = 3$.

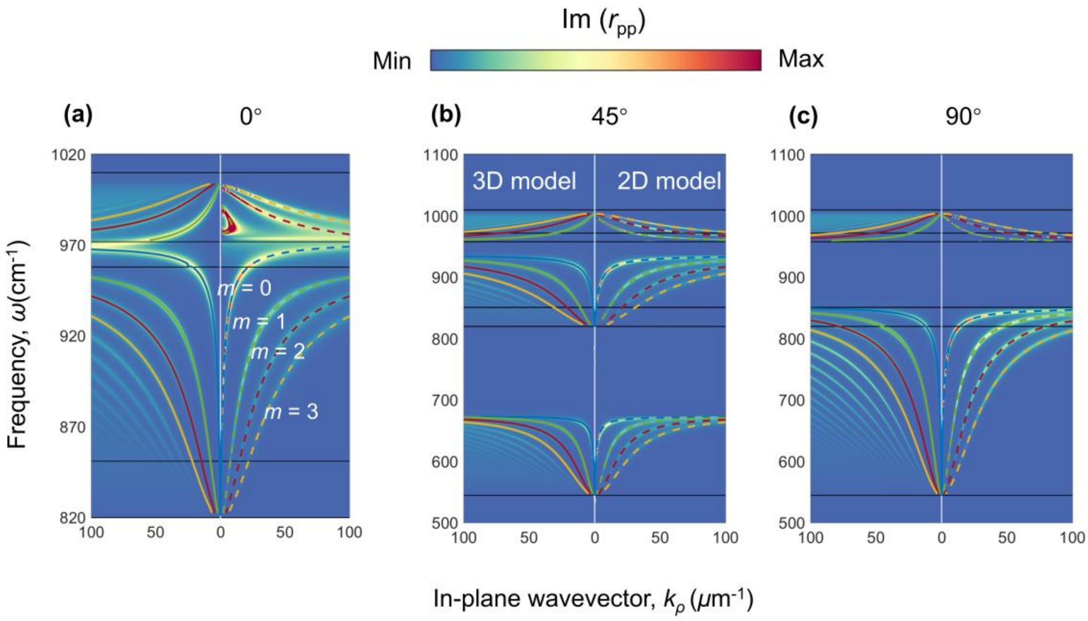

Fig. S4 The imaginary part of reflection coefficient, i.e., Im(r_{pp}) of PhPs in the α-MoO_3 plate with thickness of 200 nm for different value of φ: (a) 0°, (b) 45°, (c) 90° calculated by 3D (Left) and synthesized 2D (Right) models. The blue, green, red and yellow curves denote the $m = 0$, $m = 1$, $m = 2$, and $m = 3$ modes, respectively.

To directly compare GSCM and full 3D model, we calculate the Im(r_{pp}) distributed in the k_x-k_y momentum space as shown in Fig. S5a and S5b. Here, the thickness of α-MoO_3 slab is set to 100 nm and frequency is 900 cm^{-1}. The direct comparison of the

values of Im(r_{pp}) along the k_x axis (k_y =0) using the two models is shown in Fig. S5e. It is clearly visible that they almost completely overlap with each other, for each order polaritonic mode, which efficiently illustrates the validity of GSCM. According to Eq. 10 in the main text, the imaginary part of the trace of Green's function is associated with the LDOS. The distributions of Im(Tr(G^E)) in k_x-k_y momentum space are exhibited in Fig. S5c and S5d, which are based on synthesized GSCM and full 3D model, respectively. Their values along the k_x axis are shown in Fig. S5f where the synthesized one agrees well with the results using full 3D model. The direct comparison between synthesized GSCM and full 3D model in terms of Im(Tr(G^E)) further establishes the validity of our proposed corrected 2D model.

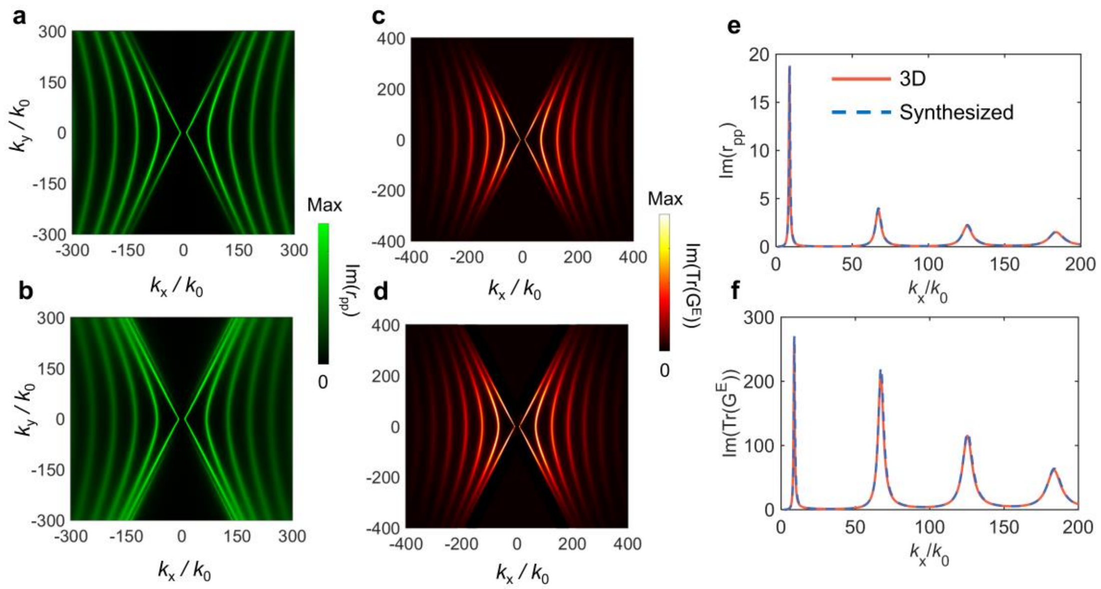

Fig. S5 The distribution of Im(r_{pp}) and Im(Tr(G^E)) in k_x-k_y momentum space calculated using (a),(c) full 3D model and (b), (d) synthesized GSCM. The α-MoO_3 plate has a thickness of 100 nm. The height of probing point is h = 10 nm. (e) and (f) compare the values of Im(r_{pp}) and Im(Tr(G^E)) based two models, respectively, as a function of k_x (k_y=0). The frequency is at ω = 900 cm^{-1}.

To corroborate the synthesized GSCM theory, we further extend the wavevector to 600 k_0 in the calculation of Im(Tr(G^E)) using GSCM and compare with the results based on full 3D model, as shown in Fig. S6a. Notably, higher-order modes have smaller

attenuation length, so that at height of 10 nm, the fundamental mode dominates the LDOS. Correspondingly, the Im(Tr(G^z)) reaches the most at wavevector of $m = 0$ mode (Fig. S6a) and higher-order modes have the smaller Im(Tr(G^z)). As the order increases, the contribution becomes weaker. When m is more than 8, their contribution to the enhancement of LDOS is negligible. That is to say, when using the synthetic GSCM, only the $m \ll 8$ PhPs mode is considered. To demonstrate this, we integrate the Im(Tr(G^E)) along the along the k_x axis for Fig.S6a, that is, $\chi = \int_0^\infty \mathrm{Im}\left(\mathrm{Tr}(G^E)\right) dk_x$. The results are shown in Fig. S6b. The blue line represents the calculation using synthetic GSCM as a function of number of polaritonic mode. The value of integration gradually increases with taking into account more polaritonic mode and reaches a stable state when the order exceeds 8.

To quantify the difference between synthetic GSCM and full 3D model, let us define the relative difference as follow:

$$\Delta_\chi = \frac{|\chi_{3D} - \chi_{2D}|}{\chi_{3D}}. \tag{S13}$$

From Fig. S6b, one can see that the relative difference can decrease to 0.043, demonstrating the validity of the GSCM.

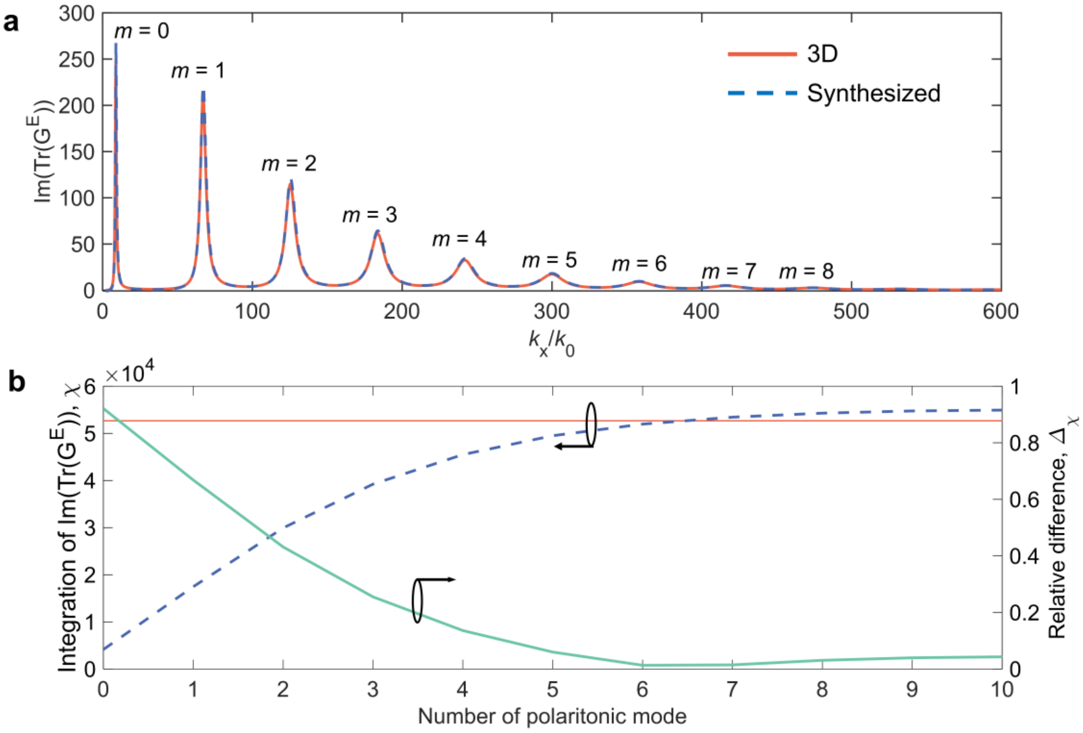

Fig. S6 (a) The value of Im(r_{pp}) calculated by synthesized GSCM (blue dashed line)

and full 3D model (orange solid line) as a function of k_x (k_y=0). (b) Left: The line integration of Im(Tr(G^E)) for Fig. S6a; Right: Relative difference between the two models. ω = 900 cm^{-1}.

Section 4: The effect of thickness of the slab on the validity of GSCM

In this section, we discuss the effect of the thickness of the slab on the validity of the corrected 2D model. For this purpose, we choose different thicknesses of α-MoO_3 plate and compare the Im(Tr(G^E)) obtained via synthesized GSCM and full 3D model, respectively, as shown in Fig. S7a-j, similar to Fig. S6a. The thickness range changes from 1 nm to 1 μm. As the thickness increases, the number of polaritonic modes included in synthesized GSCM improves to better match with the results using full 3D model. The integration of Im(Tr(G^E)) and relative difference between two models are shown in Fig. S7k. We can see that the relative difference is less than 0.065, even in the thicker configuration. One can see that the relative difference has no tendency to the thickness of slab, which means that the validity of our proposed GSCM has no evident dependence on the thickness of slab. To understand this, let's go back to the assumptions made in the derivation process. There are two of them: one is that $\frac{\rho}{\varepsilon_z} \rightarrow 0$, and the other is $k_\rho >> k_0$. That is, thickness of slab is not associated with these two assumptions. Therefore, GSCM remains effective even though the slab has a larger thickness.

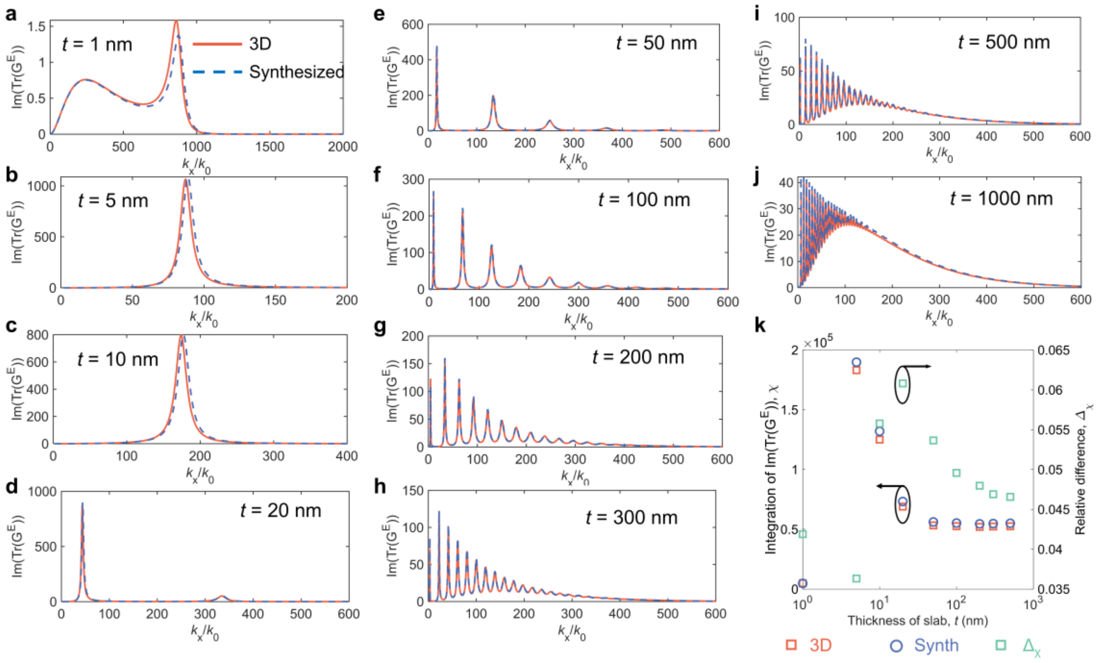

Fig. S7 Im(Tr(G^E)) calculated by synthesized GSCM and full 3D model as a function of k_x for different thicknesses of α-MoO_3 plate. (a) 1 nm; (b) 5 nm; (c)10 nm; (d) 20 nm; (e) 50 nm; (f)100 nm; (g) 200 nm; (h) 300 nm; (i)500 nm; (j) 1000 nm. (k) Left: The line integration of Im(Tr(G^E)) for Fig. S7a-j; Right: Relative difference between the two models. The frequency is fixed at ω = 900 cm^{-1}.

Section 5: The analysis of the relative difference between 2D and 3D models in dispersion

To further elucidate the effect of permittivity on the polaritonic difference, we provide a quantitative assessment of the relative difference between the 2D and 3D models, defined as

$$\Delta = \left| \frac{k_{3D} - k_{2D}}{k_{3D}} \right|. \quad \text{(S14)}$$

Here, we focus on the [100] crystal direction, i.e., x axis. The Fig. S8 shows the relative difference between two models and the value of $\frac{1}{|\varepsilon_z \varepsilon_x|}$ as a function of permittivity along the [100] crystal direction, i.e., ε_x, where the frequency is fixed to 980 cm^{-1}. One can see that the relative difference declines as the ε_x increases, which keeps the same trend of $\frac{1}{|\varepsilon_z \varepsilon_x|}$ varying with ε_x. The presence of this tendency is because that larger ε_x brings

the value of $\frac{1}{|\varepsilon_z \varepsilon_x|}$ closed to zero, making a more favorable fit to the assumption $\frac{\rho}{\varepsilon_z} \to 0$. As we know, the derivation of the corrected 2D model builds on the assumption, indicating that more the value of $\frac{1}{|\varepsilon_z \varepsilon_x|}$ converges to zero, the smaller the relative difference between these two models. The dispersion curves in the inset also show the difference between two models is correlated with the value of ε_x, where the small-ε_x curves (yellow) using 3D model and 2D model are elliptical (dashed) and bimodal-like (solid), respectively. However, as the ε_x increases, the shapes become identical and symmetrical about the *y*-axis.

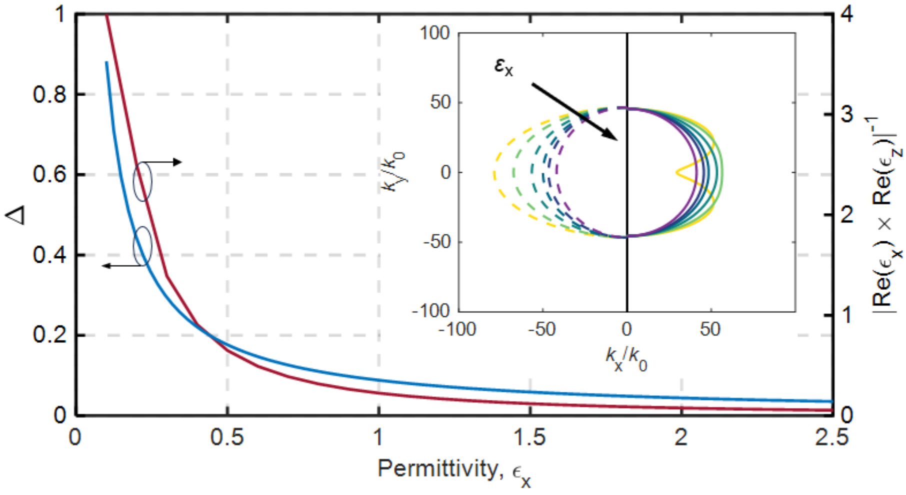

Fig. S8 Left: The relative difference between the two models defined as Eq. S13 as a function of permittivity along the [100] crystal direction, i.e., ε_x. Right: The value of $\frac{1}{|\varepsilon_z \varepsilon_x|}$. Although the frequency is fixed to 980 cm^{-1}, the permittivity ε_x is artificially changed from 0.1 to 2.5. The inset shows the comparison of analytically calculated IFCs for the 3D (Left, dashed curves) and the corrected 2D (Right, dashed curves) models. The yellow line marks the realistic value of ε_x at the frequency of 980 cm^{-1} and yellow curves are the corresponding IFCs. The thickness is 100 nm.

Section 6: The electric-field Green's function

Note that the vacuum Green's function can be neglected compared with reflection Green's function in the frequency of resonant modes. And G^E can be written as [7]

$$\mathrm{G}^{E}\left(\mathbf{r}_i,\mathbf{r}_j\right)=\frac{\mathrm{i}}{8\pi^2}\int_{-\infty}^{\infty}dk_x\int_{-\infty}^{\infty}dk_y\left(\sum_{\alpha,\beta=p,s}r_{\alpha\beta}\mathbf{a}_{\alpha}^{+}\otimes\mathbf{a}_{\beta}^{-}\right)$$
$$\times\frac{1}{k_z}\exp\left(i\left[k_x\left(x_i-x_j\right)+k_y\left(y_i-y_j\right)\right]\right)\exp\left(ik_z\left(\mathrm{z}_i+z_j\right)\right). \tag{S15}$$

Here, $r_{\alpha\beta}$ is the Fresnel reflection coefficient, which can be obtained by the TMM. The expression of polarization vectors are given as $\mathbf{a}_s^{\pm}=\frac{1}{k_\rho}\left(k_y,-k_x,0\right)^T$ and $\mathbf{a}_p^{\pm}=\frac{k_z}{k_\rho k_0}\left(\mp k_x,\mp k_y,k_\rho^2/k_z\right)^T$ where $k_\rho=\sqrt{k_x^2+k_y^2}$ and $k_z=\sqrt{k_0^2-k_\rho^2}$ represent the lateral and vertical wave vectors, respectively.

Section 7: The photonics LDOS above an h-BN slab

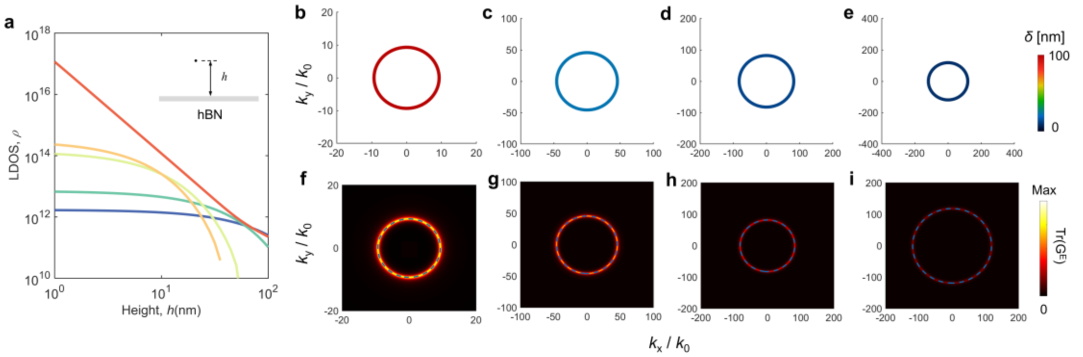

Fig. S9 (a) The LDOS as a function of height above the h-BN slab at the frequency of 1539.6 cm^{-1}. (b)-(e) The attenuation length of corresponding order mode. (f)-(i) Tr(G^{E}) for the configuration of h-BN slab at the height of 100 nm. The dashed curves correspond to the dispersion curves of phonon polaritons of m = 0 (f); m =1 (g); m = 2 (h); and m = 3 (i) modes, respectively. The thickness of h-BN slab is 100 nm.

Section 8: The calculation of electric field launched by a *z*-polarized electric dipole

In main text, the distribution of electric fields in *x*-*y* plane above a planar layered interface is calculated based on Green's function method [11], as shown in Fig. S10.

Let us first review the dipole fields in vacuum medium. In this case, the interface in Fig. S7 is removed. The dyadic Green's function $\mathbf{G}^0(\mathbf{r}_0;\,\mathbf{r})$ defines the electric field $\mathbf{E}(\mathbf{r})$ at position $\mathbf{r}$ due to an electric dipole $\mathbf{p}$ located at $\mathbf{r}_0$, according to

$$\mathbf{E}(\mathbf{r}) = \omega^2\mu_0\mathbf{G}^0(\mathbf{r}_0;\mathbf{r})\mathbf{p}. \tag{S16}$$

Further, in the presence of a substrate, the electric field at position **r** is composed of two parts: direct and reflected, which relates by the vacuum Green's function $\mathbf{G}^0(\mathbf{r}_0;\mathbf{r})$ and reflected Green's function $\mathbf{G}^R(\mathbf{r}_0;\mathbf{r})$ (i.e., G^E defined in Eq. S14). Thus, the electric field in the upper-half-space can be expressed by summing the vacuum and reflected Green's functions as

$$\mathbf{E}(\mathbf{r}) = \omega^2\mu_0[\mathbf{G}^0(\mathbf{r}_0;\mathbf{r}) + \mathbf{G}^R(\mathbf{r}_0;\mathbf{r})]\mathbf{p}. \tag{S17}$$

When using 3D model, the slab is strictly modelled as 3D structure, and the substrate is a h-BN or α-MoO_3 plate (Fig. 1d). In comparison, 2D model treats the layer as a surface without thickness, so that the substrate is a 2D surface. In so operation, Figs. 1e-h and Figs. 3c-d can be obtained.

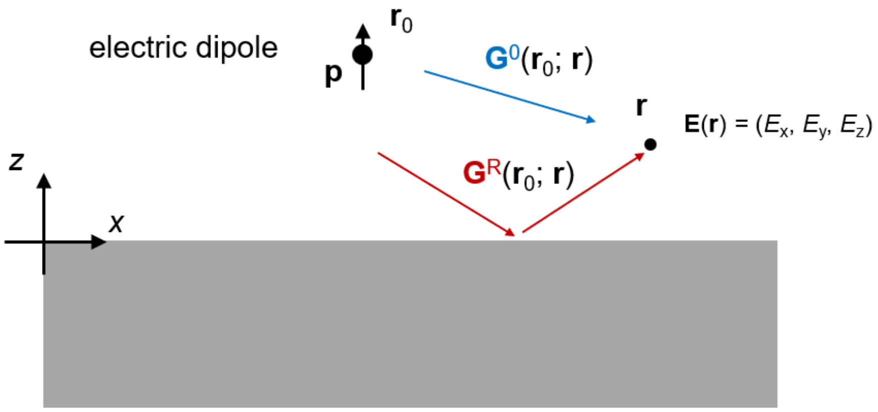

Fig. S10 Schematic of dipole-launched electric field. The electric dipole is located at $\mathbf{r}_0$, polarized along z-axis direction. The upper half-space is vacuum medium.

Section 9: The distribution of electric fields for different thicknesses using GSCM

In this section, we calculate the electric field launched by the dipole for α-MoO_3 plate with thickness of 100 and 150 nm, based on full 3D model and GSCM. The FFT of E_z calculated by corrected 2D model agrees well with the analytical dispersion curves for each order polaritonic mode. One can see that the polariton patterns of synthesized electric fields are identical to those based on 3D models. In addition, FFT of the synthesized electric field coincides with dispersion curves. These further demonstrate

the validity of GSCM.

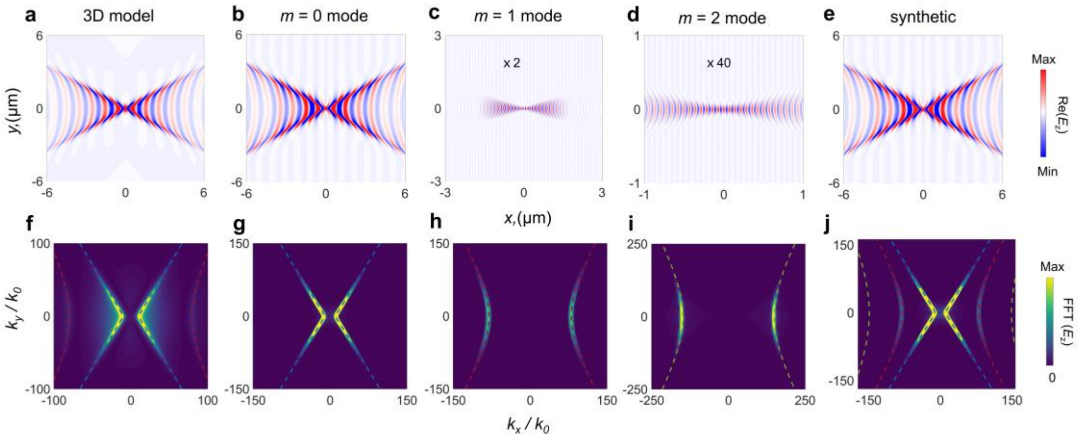

Fig. S11 Electric field distribution and dispersion of PhPs of α-MoO_3 plate with thickness of 100 nm. (a)-(d) Re(E_z) and (f)-(i) corresponding dispersion (FFT) based on 3D model and the generalized 2D model at 910 cm^{-1}. (e) The synthetic electric field, namely, the superposition of m = 0, m = 1 and m = 2. (j) FFT of the top electric field map. The dashed lines are the analytically calculated dispersion curves, among which the blue, red and green lines represent the fundamental mode, m = 1 mode, and m = 2 mode, respectively.

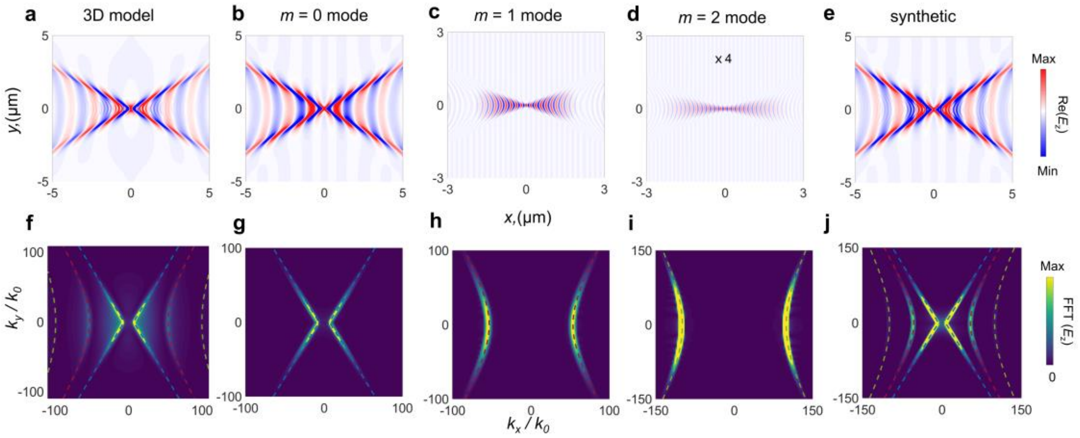

Fig. S12 Electric field distribution and dispersion of PhPs of α-MoO_3 plate with thickness of 150 nm. (a)-(d) Re(E_z) and (f)-(i) corresponding dispersion (FFT) based on 3D model and the generalized 2D model at 910 cm^{-1}. (e) The synthetic electric field, namely, the superposition of m = 0, m = 1 and m = 2. (j) FFT of the top electric field map.

Section 10: The detailed derivation of Eq. 5 in main text

The dispersion relation for a suspended slab with thickness being *d* can be written as

$$1 + r_{12}e^{i\phi} = 0, \tag{S18}$$

where r_{12} is the Fresnel reflection coefficient for *p*-polarized wave incident from medium 1, i.e., air to medium 2, i.e., *h*-BN, written as

$$r_{12} = \frac{\frac{k_{2z}}{\varepsilon_\perp} - k_{1z}}{\frac{k_{2z}}{\varepsilon_\perp} + k_{1z}}, \tag{S19}$$

Using Eq. S19, we can rewrite the Eq. S18 as

$$k_{2z} + \varepsilon_\perp k_{1z} + (k_{2z} - \varepsilon_\perp k_{1z})e^{i\phi} = 0 \tag{S20}$$

At larger wave vector, the perpendicular components of wave vector in the slab and air are expressed

$$k_{2z} = i\sqrt{\frac{\varepsilon_\perp}{\varepsilon_\parallel}}k_\rho, \tag{S21}$$

and

$$k_{1z} = ik_\rho. \tag{S22}$$

Note that we expand the term $e^{i\phi}$ into Talyor series:

$$e^{i\phi} = 1 + i(k_{2z}t + m\pi), m = 0,1,2 \ldots \tag{S23}$$

where *t* is the thickness of the vdW plate.

Therefore, substituting the Eqs. S21-S23 into Eq. S20, we can get the expression of dispersion relation. Note that the expression closely depends on the sign of $\varepsilon_\perp$.

When $\varepsilon_\perp < 0$, namely, in RB II

$$k_\rho = i\left(\frac{2}{i\left(1 - \sqrt{\varepsilon_\perp \varepsilon_\parallel}\right)} + m\pi\right)\frac{1}{t}\sqrt{\frac{\varepsilon_\parallel}{\varepsilon_\perp}} \tag{S24}$$

Comparing this with the surface dispersion relation $2 + \sigma\frac{k_{1z}}{\omega\varepsilon_0} = 0$, we get the relation between surface conductivity σ and dielectric function ε,

$$\sigma = \frac{2\omega\varepsilon_0\sqrt{\frac{\varepsilon_\perp}{\varepsilon_\parallel}}t}{\frac{2i}{\sqrt{\varepsilon_\perp\varepsilon_\parallel}} + m\pi}, \tag{S25}$$

where we simplify $\sqrt{\varepsilon_{\perp}\varepsilon_{\parallel}}-1$ as $\sqrt{\varepsilon_{\perp}\varepsilon_{\parallel}}$.

Similarly, when $\varepsilon_{\perp}>0$, namely, in RB I, we can get the relation between σ and ε

$$\sigma=\frac{2\omega\varepsilon_0\sqrt{\frac{\varepsilon_{\perp}}{\varepsilon_{\parallel}}}t}{-\frac{2i}{\sqrt{\varepsilon_{\perp}\varepsilon_{\parallel}}}+m\pi} \tag{S26}$$

References

[1] Z. Zhang, Nano/Microscale Heat Transfer. New York, NY, USA: McGraw-Hill, 2007.

[2] H. Wu et.al., Near-field radiative heat transfer modulated by nontrivial topological surface states. Materials Today Physics, 27, 100825 (2022).

[3] B. Zhao and Z. Zhang, Enhanced photon tunneling by surface plasmon–phonon polaritons in graphene/hBN heterostructures. Journal of Heat Transfer, 139(2), 022701 (2017).

[4] K. Shi, F. Bao, and S. He, Enhanced near-field thermal radiation based on multilayer graphene-hBN heterostructures. Acs Photonics, 4(4), 971-978 (2017).

[5] N. C. Passler and A. Paarmann, Generalized 4× 4 matrix formalism for light propagation in anisotropic stratified media: study of surface phonon polaritons in polar dielectric heterostructures, Journal of the Optical Society of America B, 34(10), 2128-2139 (2017).

[6] N. C. Passler, M. Jeannin, and A. Paarmann, Layer-resolved absorption of light in arbitrarily anisotropic heterostructures, Physical Review B, 101(16), 165425 (2020).

[7] S. Chen, C. Fu, and G. Hu, Phonon-Polariton-Mediated Configurable Radiative Thermal Router, ACS Photonics, 12(1), 271-281 (2024).

[8] P. Yeh. Optical Waves in Layered Media; Wiley: New York, 1988.

[9] Z. Zheng, N. Xu, S. L. Oscurato, M. Tamagnone, F. Sun, Y. Jiang, Y. Ke, J. Chen, W. Huang, W. L. Wilson, A. Ambrosio, S. Deng, and H. Chen, A mid-infrared biaxial hyperbolic van der Waals crystal, Science Advances, 5(5), eaav8690 (2019).

[10] A. Kumar, T. Low, K. H. Fung, P. Avouris, N. X. Fang, Tunable light–matter interaction and the role of hyperbolicity in graphene–hBN system, Nano Letters, 15(5), 3172-3180 (2015).

[11] L. Novotny and B. Hecht, Principles of nano-optics, 2012, Cambridge university press.
